# NFATc1-mediated activation of the pentose phosphate pathway and cell cycle dysregulation collectively drive tumor progression

**DOI:** 10.1038/s41389-025-00581-2

**Published:** 2025-11-07

**Authors:** Suyang Zhang, Guangyao Xu, Tianyu Cao, Fei Yu, Moses Okotel, Mingyue Wu, Shourong Wu, Vivi Kasim, Can Huang

**Affiliations:** 1 Metabolic Disease Research Center, Department of Biochemistry and Molecular Biology, School of Basic Medicine, Anhui Medical University, Hefei, China; 2 Stomatologic Hospital&College, Anhui Medical University, Key Lab. of Oral Diseases Research of Anhui Province, Hefei, China; 3 Key Laboratory of Biological Science and Technology, Ministry of Education, College of Bioengineering, Chongqing University, Chongqing, China

**Keywords:** Cancer metabolism, Oncogenes

## Abstract

The pentose phosphate pathway (PPP) supplies abundant reducing equivalents and biosynthetic precursors to support the rapid proliferation of tumor cells. An increased PPP flux is a hallmark of metabolic reprogramming in tumors. Although nuclear factor of activated T-cells c1 (NFATc1) promotes oncogenesis in various cancers, its role in metabolic reprogramming remains unclear. Here, we demonstrate that NFATc1 enhances NAD kinase (NADK) expression, elevating intracellular NADP^+^ levels to activate the PPP, thereby boosting proliferation. Furthermore, NFATc1 binds to both the p1 and p2 promoters of MDM2, sustaining its expression, thereby promoting metabolic reprogramming and accelerating cell cycle progression. Finally, we demonstrated that NFATc1 inhibitors suppress colorectal cancer (CRC) growth by targeting the NFATc1/NADK and NFATc1/MDM2 axis and synergize with oxaliplatin. In summary, our findings reveal that targeting NFATc1 simultaneously restricts biosynthetic precursors and impairs cell cycle progression in CRC, suggesting that NFATc1 inhibition is a promising therapeutic strategy.

## Introduction

Colorectal cancer (CRC) accounts for approximately 10% of all cancer cases and is the second leading cause of cancer-related deaths [1, 2]. Despite recent advancements in medical interventions, including surgery, radiotherapy, and systematic therapy, there has been no significant improvement in the five-year survival rate of patients with CRC [3, 4].

Cancer metabolic reprogramming, a hallmark of malignancy, is characterized by profound alterations in cellular metabolism that support uncontrolled proliferation, stress adaptation, and metastatic dissemination [5]. Originally conceptualized by Otto Warburg in the 1920s through his groundbreaking observation of aerobic glycolysis, cancer cells prefer lactate-producing glycolysis over oxidative phosphorylation (OXPHOS), even in the presence of oxygen, which involves multiple interconnected pathways [6]. The pentose phosphate pathway (PPP) plays a pivotal role as a critical facilitator of tumorigenesis [7]. PPP activation increases NADPH production and generates ribose-5-phosphate (R5P). NADPH overproduction plays a critical role in maintaining redox homeostasis, driving lipid biosynthesis, and supplying amino acids to facilitate cell proliferation. Concurrently, R5P acts as a rate-limiting substrate for nucleotide biosynthesis, directly supporting DNA replication and transcriptional activity during rapid cell proliferation [8, 9]. However, given its complex regulatory network, the underlying regulatory mechanism remains elusive and requires further investigation to provide additional insights into targeted tumor metabolism for cancer therapy [10].

NFATc1, a member of the NFAT transcription factor family, was originally identified as a key regulator of immune cell activation, particularly in T-cell signaling pathways [11–13]. Previous studies have established that NFATc1 overexpression is strongly associated with aggressive tumor behavior and poor clinical outcomes in various cancers, including pancreatic, gastric, and colon cancer [14–16]. However, their roles in metabolic reprogramming remain unclear. Although recent research has shown that NFATc1 enhances glycolytic metabolism to fuel gastric cancer progression [17], its broader impact on other critical metabolic pathways, particularly the PPP, remains elusive.

In this study, we identified NFATc1 as a novel transcriptional regulator of nicotinamide adenine dinucleotide kinase (NADK) using a comprehensive multi-database analysis. As a pivotal metabolic enzyme, NADK catalyzes the phosphorylation of NAD⁺ to generate NADP⁺. Both molecules serve as essential cofactors for oxidoreductases in cellular metabolism [18, 19]. Given their central role in redox reactions, perturbations in NADK-mediated NADP^+^ production can significantly alter the metabolic flux [20]. Our findings demonstrate that NFATc1 enhances NADK transcriptional activity by directly binding to its promoter region, thereby upregulating NADK expression. Increased NADK expression markedly elevates NADP⁺ levels, thereby reducing NAD⁺ levels. This altered NAD⁺/NADP⁺ ratio promotes an increased flux through the PPP. This metabolic reprogramming promotes the generation of downstream metabolites that serve as essential substrates for colon cancer cell proliferation. Interestingly, we observed that the proliferative impairment caused by NFATc1 knockdown could not be completely rescued by NADK overexpression. Further investigations revealed that NFATc1 simultaneously regulates both NADK and MDM2, which collectively promote metabolic reprogramming and cell cycle progression, thereby accelerating cancer cell proliferation. This dual regulatory mechanism suggests that pharmacological inhibition of NFATc1 could provide a two-pronged therapeutic approach to suppress colon cancer growth.

Our study elucidates a novel mechanism by which NFATc1 reprograms tumor metabolism and proposes a promising antitumor strategy targeting this pivotal regulatory pathway.

## Materials and Methods

### Vector construction

shRNA expression vectors were constructed as described previously [21]. The specific target sites for *NFATc1* were GCTTGGGCCTGTACCACAA, GAGGAAGAACACACGGGTA, and AGCAGAGCACGGACAGCTA, respectively. The specific target sites for *p53* were GCAAGAAGGGAGACAAGAT. For *NFATc1* and *NADK* overexpression vectors (pcNFATc1), human cDNA was obtained by reverse-transcribing total RNA extracted from HCT116 cells using the FastPure Cell/Tissue Total RNA Isolation Kit V2 (Vazyme, Nanjing, China). The corresponding regions were then amplified from the cDNA using the Takara Prime STAR Max DNA Polymerase (Takara Bio, Dalian, China). The *NFATc1* CDS was directionally cloned between the Hind III and BamH I restriction sites of pcDNA3.1^+^, and the *NADK* CDS was subcloned into the Kpn I and Xho I sites of pcDNA3.1-6×His.

MDM2 and p53 expression vectors were constructed as described previously [22].

For *NADK* and *MDM2* promoter *p1* and *p2* luciferase reporter vector, the corresponding promoter regions were amplified using the Takara PrimeSTAR Max DNA Polymerase (Takara) from human genome DNA extracted from HCT116 cells using the TIANamp Genomic DNA Kit (Tiangen Biotech, Beijing, China) and cloned into the Bgl II and Hind III sites of pGL4.13 vector (Promega, Madison, WI). Utilizing the Site-directed Mutagenesis Kit (Beyotime Biotechnology), the luciferase reporter vector with modified NFATc1 binding sites was created.

### Cell cultures and cell lines

HCT116 wild type CRC cell lines, the HGC-7901 human gastric carcinoma cell lines were obtained from the Cell Bank of the Chinese Academy of Sciences (Shanghai, China). HCT116^p53 null^ cell lines were provided by B. Vogelstein (Johns Hopkins University School of Medicine). HT29 human CRC cell lines were provided by H. Zhang (Anhui medical university).

HCT116 cell lines were cultured in McCoy’s 5 A medium (Gibco, Life Technologies, Grand Island, NY) supplemented with 10% fetal bovine serum (Biological Industries, Beit Haemek, Israel). The HT29 human CRC cell line were cultured in DMEM medium (Gibco) supplemented with 10% fetal bovine serum. The HGC-7901 human gastric carcinoma cell line were cultured in 1640 medium (Gibco) supplemented with 10% fetal bovine serum. Cell lines were verified using short-tandem repeat profiling method and were tested periodically for mycoplasma contamination using Mycoplasma Detection Kit-QuickTest (Biotool, Houston, TX).

Gene knockdown, overexpression, co-transfection experiments and construct gene knockdown stable cells linewere performed as previously described [22].

For NFATc1 inhibitor NFAT-IN-1(NFAT-IN) (MedChemExpress, Monmouth Junction, NJ) and NIFE (Selleckchem, Houston, TX) treatment, 1 × 10^6^ cells were seeded in 6-well plate and cultured for 24 h, then the cells were treated with NFAT-IN or NIFE (final concentration: 10 μM).

For oxaliplatin (MedChemExpress, Monmouth Junction, NJ) treatment, 1×10^6^ cells were seeded in 6-well plate and cultured for 24 h, then the cells were treated with oxaliplatin (final concentration: 10 μM).

### Xenograft experiment

For the in vivo tumor study (*n* = 5), male BALB/c-nu/nu mice (weight, 18–22 g; 6 weeks old) were obtained from the Laboratory Animal Center of Anhui Medical University (Hefei, China) and randomly divided into several groups. All animal experiments conformed to the approved guidelines of the Animal Care and Use Committee of the Anhui Medical University. All efforts to minimize suffering were made. The investigator was blinded to the group allocation and during the assessment. Xenograft experiment was performed as previously described [22].

### Tumor treatment

6 days after injection, mice with tumor received a treatment of oxaliplatin at a dose of 10 mg.kg^−1^ every three days (i.p.), NFAT-IN at a dose of 10 mg.kg^−1^ once daily (i.p.) and NIFE at a dose of 0.1 mg.kg^−1^ three times a day (gavage). Tumor size was measured every 3 days.

### Clinical human CRC specimens

Human CRC specimens were obtained from CRC patients undergoing surgery at The First Affiliated Hospital of Anhui Medical University (Hefei, China). Patients did not receive chemotherapy, radiotherapy or other adjuvant therapies prior to the surgery. Prior patient’s written informed consents were obtained, and the experiments were approved by the Institutional Research Ethics Committee of Anhui Medical University (Approval No. 20210518), and conducted in accordance with Declaration of Helsinki.

### Immunohistochemical analysis

Immunohistochemical analysis was performed as previously [23]. Briefly, the tissue sections were incubated with primary antibodies for 1 h and secondary antibodies 1 h. Visualization was performed using a DAB Kit (Agilent DAKO, Shanghai, China) under microscope. The nuclei were then counterstained with hematoxylin. Images were taken using NanoZoomer digital Pathology.

### RNA extraction and quantitative RT-PCR (qRT-PCR) analysis

Total RNAs were extracted using FastPure Cell/Tissue Total RNA Isolation Kit V2 (Vazyme) according to the manufacturer’s instruction. Each total RNA sample (1 μg) was reverse-transcribed into cDNA using the HiScript III 1st Strand cDNA Synthesis Kit ( + gDNA wiper) (Vazyme), and qPCR was performed to assess the mRNA expression levels with ChamQ Universal SYBR qPCR Master Mix (Vazyme). The sequences of the primers used for qPCR were shown in Supplementary Table S1. β-Actin was used to normalise sample amplification.

### Western blotting analysis

Western blot was performed as described previously [24]. Briefly, Samples with equal amounts proteins were electrophoresed on sodium dodecyl sulphate polyacrylamide gel before being transferred to a polyvinylidene fluoride membrane (Millipore, Shanghai, China) with 0.45 μm pore size. The antibodies used were listed in Supplementary Table S2, and immunoblotting with anti-β-Actin or GAPDH antibody was conducted to ensure equal protein loading. The signals were measured using Hypersensitive ECL Chemiluminescence Detection Kit (Proteintech, Wuhan, China). Images of uncropped blots are shown in Supplemental data-the full length uncropped original western blots.

### Intracellular NAD(H) and NADP(H) level

5 × 10⁶ cells were harvested, washed with PBS, and lysed via ultrasonic disruption. NAD^+^ levels were quantified using a Coenzyme I NAD (H) Content Assay Kit (Boxbio, Beijing, China) according to the manufacturer’s protocol. Both NADPH and NADP^+^ levels were quantified using a Coenzyme II NADP(H) Content Assay Kit (Boxbio, Beijing, China) according to the manufacturer’s protocol.

### ROS staining

2 × 10⁵ cells were seeded in 12-well plates and allowed to adhere for 24 h. The fluorescent probe DCFH-DA (Reactive Oxygen Species Assay Kit, Beyotime Biotechnology) was loaded into cells according to the manufacturer’s protocol. Images were captured by using a Zeiss AxioImager microscope.

### Immunofluorescence staining

Immunofluorescence staining was performed according to the standard protocol described previously [23]. The antibodies used were listed in Supplementary Table S2, and the nuclei were stained with DAPI.

### Statistical analysis

All quantification results were presented as mean ± SD (*n* = 3; unless otherwise indicated). Statistical analysis was performed using GraphPad Prism version 8.0 software. The difference between two groups was calculated using unpaired student t-test. Differences among multiple groups were calculated using two-way ANOVA. Values of *p < 0.05 were considered to indicate statistical significance.

## Results

### NFATc1 promotes CRC cell tumorigenic potential

**Fig. 1:**
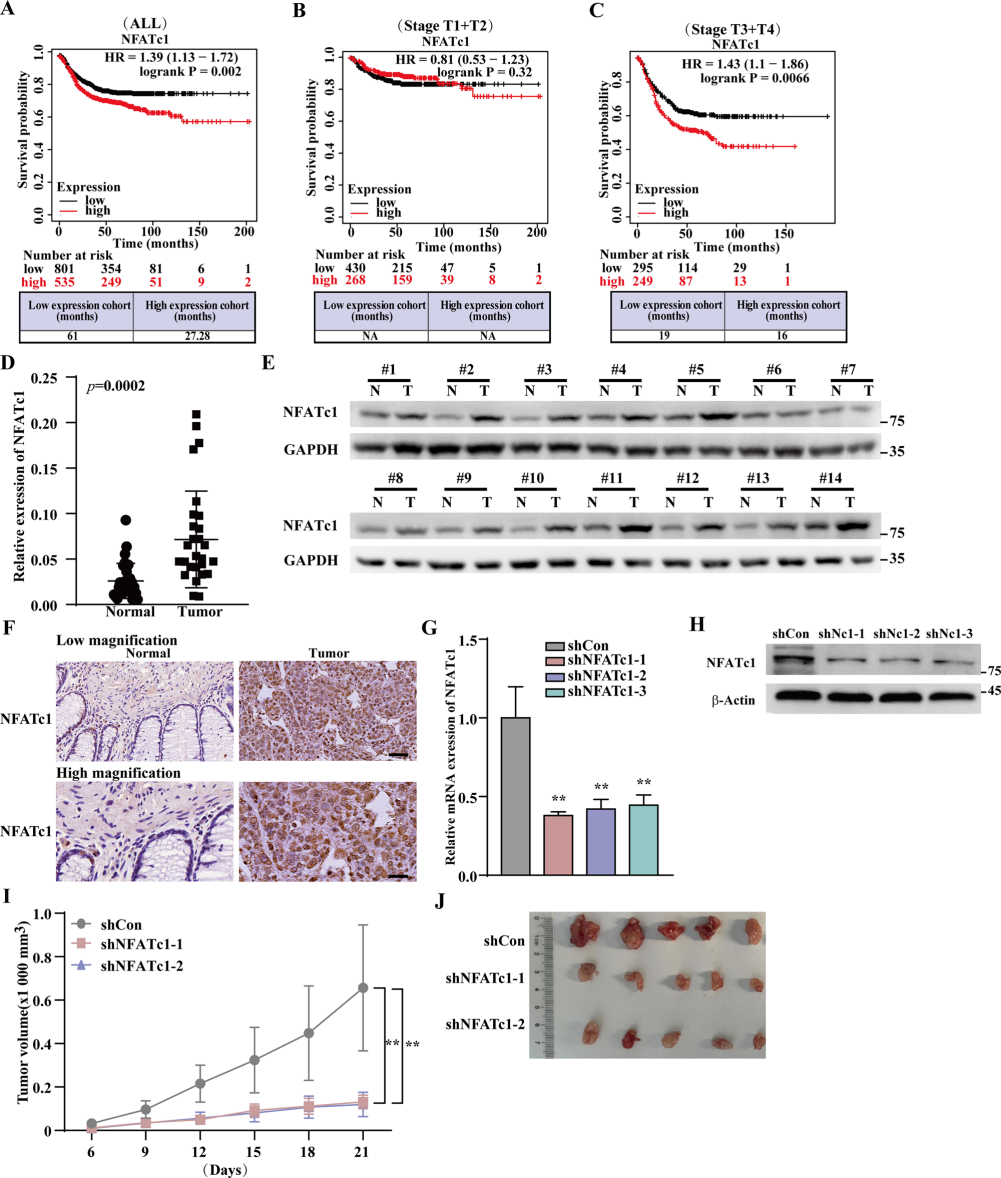
NFATc1 was positively correlated with CRC malignancy. **A** Survival curve of all CRC patients with either low (blue) or high (red) NFATc1 expression (*n* = 1336) using KM plotter (https://kmplot.com/analysis/#). **B**, **C** Survival curve of CRC patients in stage T1 and T2 (*n* = 698), T3 and T4 (*n* = 544). **D** NFATc1 mRNA expression level in clinical human colorectal carcinoma and corresponding adjacent tissue samples (*n* = 30), as determined by quantitative RT-PCR (qPCR). **E** NFATc1 protein expression level in clinical human colorectal carcinoma and corresponding adjacent tissue samples. **F** Immunohistochemistry staining showing the expression levels and localization of NFATc1 in clinical tissue. Representative of low-magnification (scale bars, 40 μm) and high magnification (scale bars, 20 μm) images. **G**, **H** NFATc1 mRNA expression and protein level in HCT116 cells transfected with shRNA expression vectors targeting 3 different sites of NFATc1, as determined using qPCR. I-J Tumor volume (**I**) and representative morphological images (**J**) of the BALB/c-nu/nu mice transplanted subcutaneously with HCT116/shCon, HCT116/shNFATc1-1and HCT116/shNFATc1–2 stable cell lines at 21 days post-transplantation (*n* = 5). Cells transfected with control vector shCon were used as control. β-Actin was used for qPCR normalization and as western blotting loading control. Quantitative data were expressed as mean ± SD. **p < 0.01; Nc1: NFATc1.

To investigate the role of NFATc1 in CRC, we analyzed the correlation between NFATc1 expression and the survival of patients with CRC using a Kaplan-Meier plotter (https://kmplot.com/analysis). The results revealed a significant correlation between high NFATc1 expression and poor survival outcomes in patients with CRC (Fig. 1A). Moreover, this correlation was stronger in advanced clinical stages (Fig. 1B, C). NFATc1 expression was also associated with poor prognosis in patients with gastric and lung cancers (Fig. S1A, S1B).

Next, we investigated NFATc1 expression in clinical samples obtained from patients with untreated CRC. NFATc1 expression was upregulated in CRC lesions compared to that in normal adjacent tissues (Fig. 1D, E). Immunohistochemical staining further confirmed its increased expression and nuclear localization in CRC lesions compared to that in normal adjacent tissues (Fig. 1F). These results suggest that high NFATc1 expression is correlated with tumorigenesis and poor prognosis.

To confirm the effects of NFATc1 on CRC, we used three shRNA expression vectors targeting different *NFATc1* sites (Fig. 1G, H). Based on these suppressive effects, we selected shNFATc1-1 and shNFATc1-2 for subsequent experiments. We performed xenograft experiments by transplanting HCT116^shNFATc1-1^ or HCT116^shNFATc1-2^ stable cells. Consistent with the findings from clinical samples, our results demonstrated that *NFATc1* knockdown significantly reduced the tumorigenic potential of HCT116 cells (Fig. 1I, J). Collectively, these results indicate that NFATc1 is crucial for the tumorigenic potential of CRC cells.

### NFATc1 promotes NADK expression and induces metabolic reprogramming in colon cancer cells

**Fig. 2:**
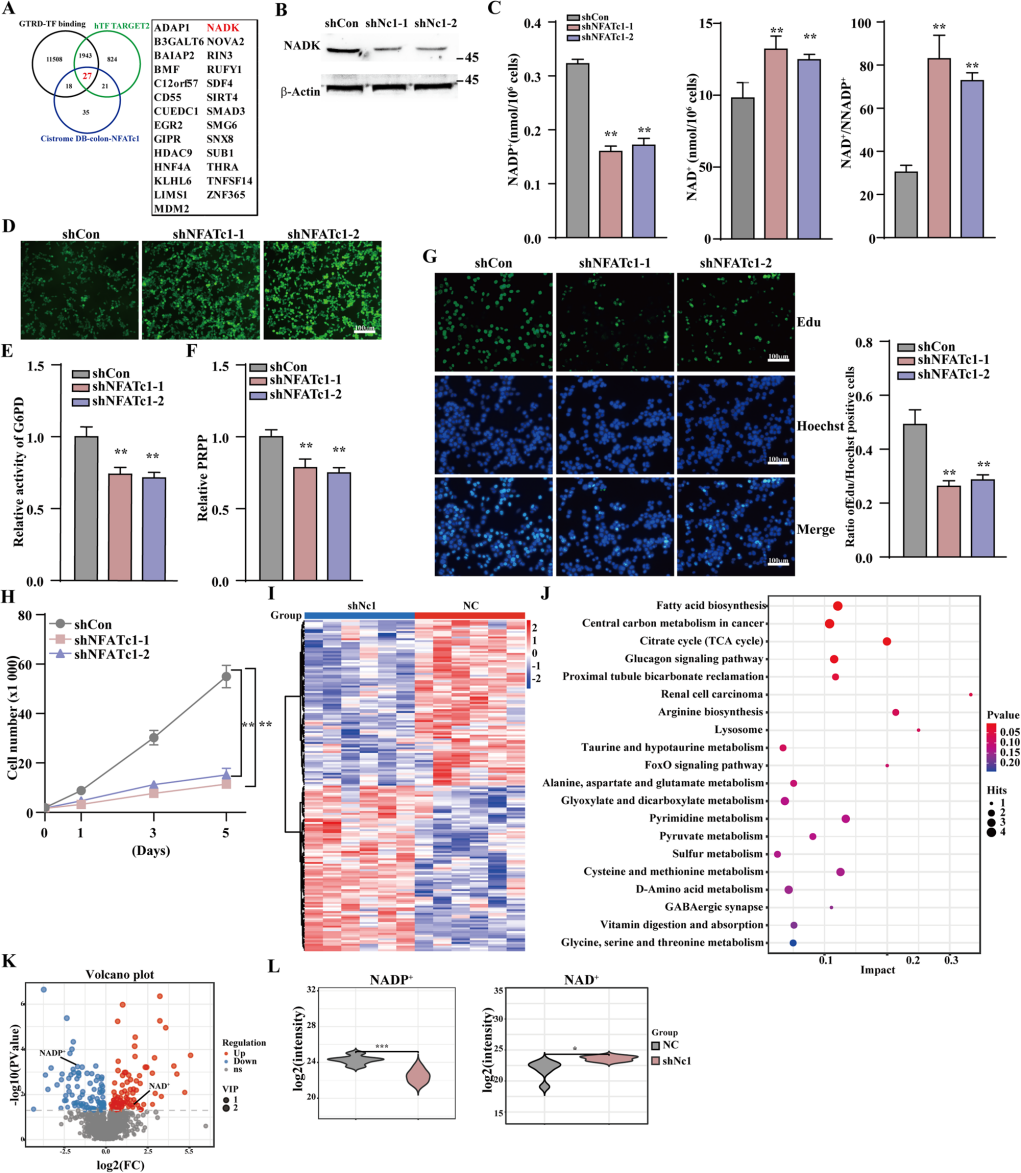
NFATc1 enhances the pentose phosphate pathway and stimulates CRC cell proliferation. **A** Potential target genes of NFATc1. **B** NADK protein expression level in HCT116 cells transfected with shRNA expression vectors targeting different sites of NFAFc1, as determined by western blotting. **C** The levels of NADP^+^, NAD^+^ and the NAD^+^ to NADP^+^ ratio, following NFATc1 knockdown. **D** ROS levels in HCT116 cells upon NFATc1 knockdown (scale bars, 100 μm). **E** Relative G6PD activity in HCT116 cells upon NFATc1 knockdown. **F** The levels of PRPP following NFATc1 knockdown. **G** Representative images of proliferating cells, as identified by the EdU incorporation assay. Hoechst was used to stain nuclei (scale bars, 100 μm). **H** Total cell number of NFATc1-knockdown HCT116 cells were measured at the indicated time. **I** Clustered heatmap of differential metabolites following NFATc1 knockdown. **J** Bubble plot depicting the regulatory impact of nfatc1 knockdown on metabolic pathways. **K** Volcano plot of differential metabolites after NFATc1 knockdown. **L** Violin plots quantifying NAD⁺ and NADP⁺ levels following NFATc1 inhibition. Cells transfected with shCon vector were used as control. β-Actin was used for western blotting loading control. Quantitative data were expressed as mean ± SD. **p < 0.01; Nc1 NFATc1.

To systematically identify NFATc1-regulated downstream targets, we conducted an integrative analysis of three complementary transcriptional databases (GTRD-TF binding, hTF TARGET2, and Cistrome DB) and identified 27 genes that consistently overlapped across all three datasets (Fig. 2A). NADK has emerged as a high-priority candidate with tumor metabolism-related characteristics. We investigated the correlation between NFATc1 and NADK in clinical colon cancer samples using data from the TCGA database. Our analysis revealed a significantly positive correlation between these two factors (*p* < 0.001) (Fig. S2A). Furthermore, this correlation was consistently observed in both gastric (*p* < 0.001) and lung (*p* = 0.009) cancer tissues (Fig. S2B, S2C). We demonstrated that the mRNA and protein expression levels of NADK significantly decreased following NFATc1 knockdown (Fig. 2B, S2D). This suppression led to a reduction in intracellular NADP⁺ levels while simultaneously elevating NAD⁺ levels, ultimately causing a marked increase in the NAD⁺/NADP⁺ ratio (Fig. 2C). Furthermore, we observed a concomitant decrease in NADPH levels, leading to a significant increase in the NADPH/NADP⁺ ratio (Fig. S2E). As NADPH plays a crucial role in cellular redox homeostasis by serving as a key reducing equivalent for antioxidant systems and effectively scavenging reactive oxygen species (ROS), we measured ROS levels and found that NFATc1 knockdown resulted in a significant increase in ROS (Fig. 2D). Given that the NADPH/NADP^+^ ratio serves as a key allosteric regulator of G6PD, the rate-limiting enzyme of the PPP, we subsequently assessed and observed a significant reduction in G6PD activity following NFATc1 suppression (Fig. 2E). This enzymatic impairment leads to attenuated PPP flux, resulting in markedly decreased production of phosphoribosyl pyrophosphate (PRPP), an essential precursor for de novo nucleotide biosynthesis (Fig. 2F). Consequently, this metabolic perturbation substantially inhibited cell proliferation (Fig. 2G, H).

To further validate the metabolic alterations resulting from NFATc1 downregulation, we performed untargeted metabolomics analysis. The results showed that suppression of NFATc1 led to consistent and significant alterations in 182 metabolites (Fig. 2I, Table S3). Pathway enrichment analysis revealed that “Central carbon metabolism in cancer” was among the most prominently altered pathways. In addition, metabolites involved in fatty acid biosynthesis, nucleotide metabolism, and amino acid metabolism were also highly enriched (Fig. 2J). Stable metabolic alterations are illustrated in the volcano plot in Fig. 2K, L. Notably, NAD⁺ levels increased significantly upon NFATc1 knockdown, while NADP⁺ levels markedly decreased. Additionally, a pronounced reduction was observed in the synthesis of R5P and multiple phospholipids (Table S3). These findings further support that the disruption of the NAD⁺/NADP⁺ balance caused by NFATc1 inhibition reduces the flux through the PPP, thereby decreasing the production of biosynthetic precursors essential for new cell generation and ultimately inhibiting tumor cell proliferation.

### NFATc1 enhances NADK transcription by directly binding to its promoter region

**Fig. 3:**
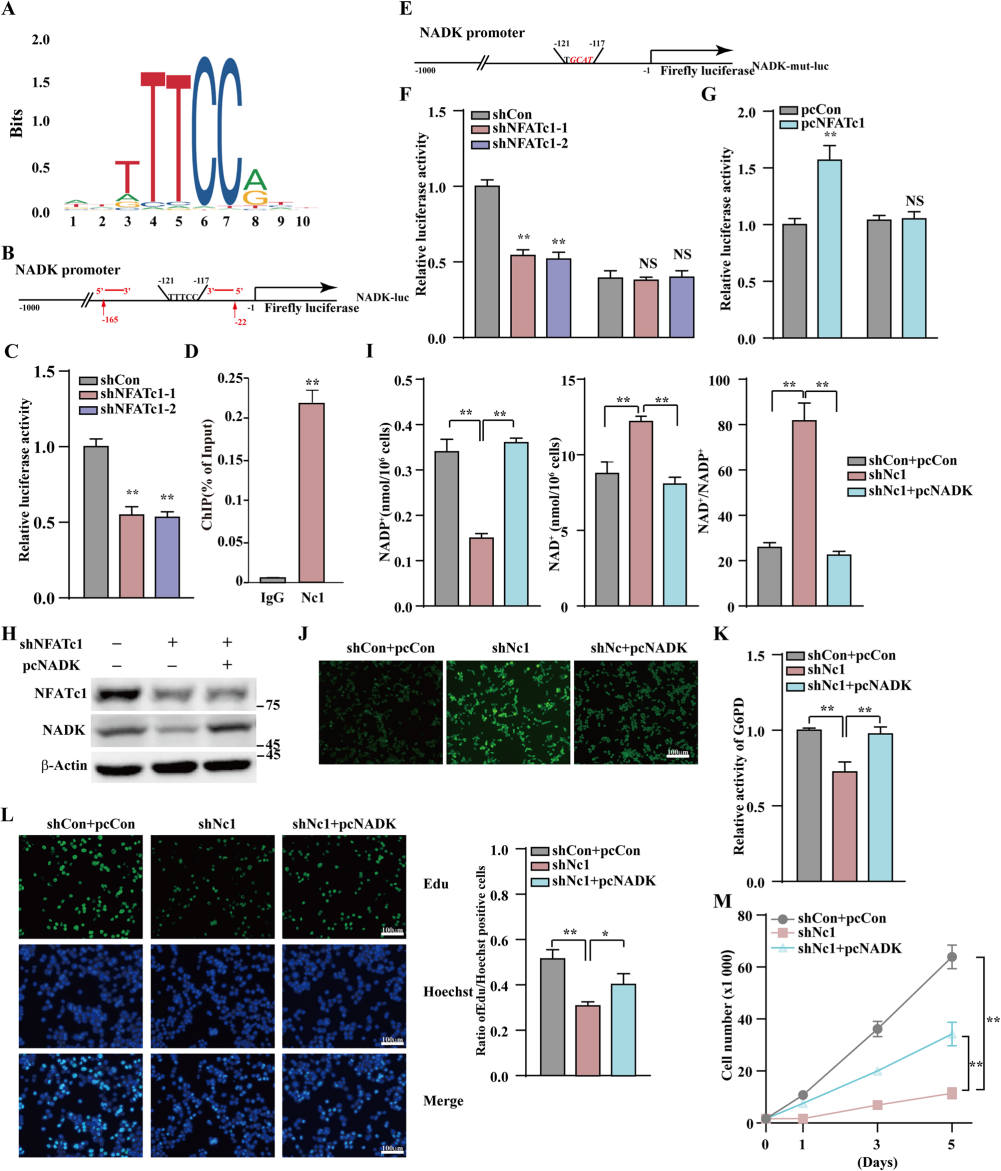
NFATc1 enhances the pentose phosphate pathway by promoting NADK transcription. **A** Predicted NFATc1 binding site from JASPAR database. **B** Schematic diagram of promoters of NADK. **C** Relative luciferase reporter activity of the NADK promoter. **D** Binding of NFATc1 to the NADK promoter region was examined using chromatin immunoprecipitation assay with anti-NFATc1 antibody followed by qPCR in HCT116 cells. The predicted NFATc1 binding site on the NADK promoter and the location of primer sets used for qPCR for amplifying NADK promoter region with NFATc1 binding site are shown in (**B**). **E** Schematic diagram of NADK promoter reporter vector with mutated NFATc1 binding sites. Those mutated nucleotides are shown in italic red. **F**, **G** Effects of NFATc1 knockdown (**F**) and overexpression (**G**) on the relative luciferase activity of NADK promoter and mutated NADK promoter. **H** Protein expression level of NFATc1 and NADK in HCT116 cells transfected with indicated shRNA expression vectors and NADK overexpression vectors. **I** NADP⁺, NAD⁺ and NAD⁺/NADP⁺ ratio in HCT116 cells following NFATc1 knockdown and NADK overexpression. **J** ROS levels in HCT116 cells following NFATc1 knockdown and NADK overexpression (scale bars, 100 μm). **K** Relative G6PD activity in HCT116 cells following NFATc1 knockdown and NADK overexpression. **L** Representative images of proliferating cells, as identified by the EdU incorporation assay. Hoechst was used to stain nuclei (scale bars, 100 μm). **M** Total cell number was assessed in NFATc1-knockdown and NADK-overexpressing HCT116 cells at indicated time. Cells transfected with shCon were used as controls. β-Actin was used for western blotting loading control. Quantitative data were expressed as mean ± SD. *p < 0.05; **p < 0.01; NS not significant, Nc1 NFATc1.

To elucidate the molecular mechanism by which NFATc1 regulates *NADK* transcription, we searched for the DNA-binding motif of NFATc1 using the JASPAR database (http://jaspar.genereg.net/); however, there was no predicted motif of NFATc1 in *Homo sapiens* DNA. Nonetheless, we noted that other NFAT family members, NFATc2 and NFATc3, share a similar DNA-binding motif with *Homo sapiens* DNA (TTTCC; Fig. 3A). NFAT family proteins share a moderately conserved N-terminal homologous region that includes a transactivation domain [11]. Therefore, we explored whether NFATc1, as a transcription factor, binds to this motif. Based on the JASPAR database predictions, we identified a putative binding site located at positions -121 to -117 (Fig. 3B). NFATc1 knockdown significantly reduced the transcriptional activity of the NADK promoter (Fig. 3C). Consistently, in CRC HT29 and gastric cancer HGC-7901 cell lines, NFATc1 knockdown significantly reduced the transcriptional activity of NADK (Fig. S3A, B). Subsequently, we performed a chromatin immunoprecipitation assay, which confirmed that NFATc1 bound to the −165 to −22 region of the *NADK* promoter (Fig. 3D). A luciferase reporter assay using NADK-mut-luc, an NADK-luc reporter with four mutated nucleotides in the predicted NFATc1-binding site (TTCC to GCAT), demonstrated that mutations in the binding site abolished the suppressive effect of NFATc1 knockdown and enhanced the effect of NFATc1 overexpression compared to that of wild-type NADK-luc (Fig. 3E–G). These results demonstrate that NFATc1 binds to the *NADK* promoter, thereby upregulating its transcriptional activity.

To investigate NADK’s role in NFATc1-mediated metabolic reprogramming and cell proliferation, we co-transfected an NFATc1 shRNA expression vector with an NADK overexpression vector (Fig. 3H). NADK overexpression reversed the NFATc1 knockdown-induced reduction in NADP⁺ levels and elevation in NAD⁺ levels, thereby restoring the NAD⁺/NADP⁺ ratio to baseline (Fig. 3I). Notably, NADK reconstitution reversed the NFATc1 knockdown-induced alterations in ROS homeostasis and G6PD activity (Fig. 3J, K), but failed to fully recover the decline in cell proliferation and viability (Fig. 3L, M). The observed effects indicated that growth inhibition mediated by NFATc1 knockdown could not be fully explained by NADK downregulation alone, implying the involvement of additional proliferation-related signaling pathways.

### NFATc1 promotes CRC cell proliferation by concurrently activating both NADK and MDM2 signaling pathways

**Fig. 4:**
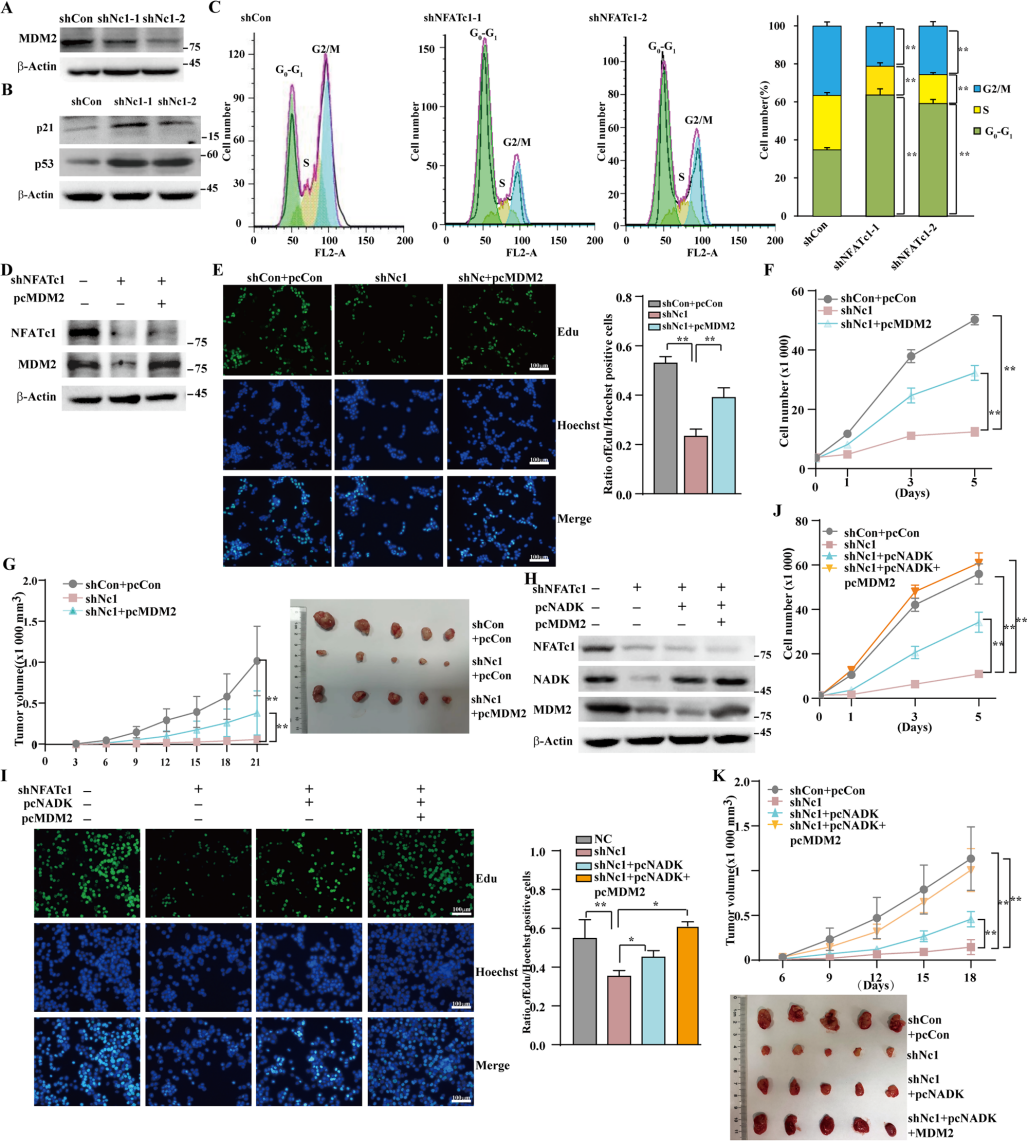
NFATc1 enhances the proliferation of CRC cells through activation of NADK and MDM2 transcription. **A** MDM2 protein expression level in HCT116 cells transfected with shRNA expression vectors targeting different sites of NFAFc1, as determined by western blotting. **B** p21 and p53 protein expression level in HCT116 cells transfected with shRNA expression vectors targeting different sites of NFAFc1, as determined by western blotting. **C** Different cell cycle phases of NFATc1-silenced HCT116 cells. Representative images are shown, and the average percentage of cells in each cell cycle phase from three independent experiments was calculated. **D** Protein expression level of NFATc1 and MDM2 in HCT116 cells transfected with indicated shRNA expression vectors and MDM2 overexpression vectors. **E** Representative images of proliferating cells, as identified by the EdU incorporation assay. Hoechst was used to stain nuclei (scale bars, 100 μm). **F** Total cell number was assessed in HCT116 cells following NFATc1 knockdown and MDM2 overexpression at the indicated time points. **G** Tumor volume and representative morphological images of the BALB/c-nu/nu mice transplanted subcutaneously with indicated stable cell lines. **H** Protein expression level of NFATc1, NADK, and MDM2 in HCT116 cells transfected with indicated shRNA expression vectors and NADK/MDM2 overexpression vectors. **I** Representative images of proliferating cells, as identified by the EdU incorporation assay. Hoechst was used to stain nuclei (scale bars, 100 μm). **J** Total cell number was assessed in HCT116 cells following NFATc1 knockdown and NADK/MDM2 overexpression at the indicated time points. **K** Tumor volume and representative morphological images of the BALB/c-nu/nu mice transplanted subcutaneously with indicated stable cell lines. Cells transfected with shCon and pcCon vector were used as control. β-Actin was used as western blotting loading control. Quantitative data were expressed as mean ± SD. *p < 0.05; **p < 0.01; Nc1 NFATc1.

Bioinformatics analysis predicted MDM2 as a potential transcriptional target of NFATc1 (Fig. 2A). Notably, NFATc1 expression was significantly and positively correlated with MDM2 levels in multiple cancer types, including CRC, gastric cancer, and lung cancer (Fig. S4A–C). As a key oncogene, MDM2 promotes cancer cell proliferation and cell cycle progression [17, 25]. In conjunction with our previous findings, these results suggest that NFATc1 knockdown concurrently reduces the expression of NADK and MDM2, leading to decreased levels of metabolic precursors and impaired cell cycle progression. Simultaneous restoration of NADK and MDM2 may be essential for reversing the reduction in cell proliferation caused by NFATc1 knockdown. Therefore, we first verified the inhibitory effect of NFATc1 knockdown on MDM2 expression (Fig. 4A and S4D) and the subsequent increase in p53 and p21 levels (Fig. 4B). Cell cycle analysis revealed that NFATc1 knockdown significantly arrested cell cycle progression (Fig. 4C). Through rescue experiments combining NFATc1 knockdown with MDM2 overexpression (Fig. 4D), we observed a partial reversal of the NFATc1 knockdown-induced suppression of both cell proliferation (Fig. 4E, F) and tumor growth (Fig. 4G). Furthermore, it has been reported that MDM2 can promote tumor metabolic reprogramming by enhancing glucose uptake or directly activating pathways such as G6PD [26–28]. Our results further support this notion, demonstrating that overexpression of MDM2 significantly rescues the decrease in glucose consumption and G6PD activity induced by NFATc1 knockdown. This finding suggests that the NFATc1/MDM2 pathway may act synergistically with the NFATc1/NADK axis to activate the PPP, thereby promoting the production of biosynthetic precursors essential for tumor cell proliferation. To further validate this cooperative mechanism, we overexpressed both NADK and MDM2 under conditions of NFATc1 knockdown (Fig. 4H). This combined intervention resulted in a more robust rescue effect: it not only fully reversed the suppression of cell proliferation induced by NFATc1 deficiency (Figs. 4I, J) but also completely restored the impaired tumorigenic capacity resulting from NFATc1 knockdown (Fig. 4K). These findings collectively establish NFATc1 as a key transcriptional regulator that simultaneously upregulates both NADK and MDM2, thereby coordinately enhancing tumor cell metabolic activity and proliferative capacity.

### NFATc1 promotes MDM2 expression by directly binding to both p1 and p2 promoter

**Fig. 5:**
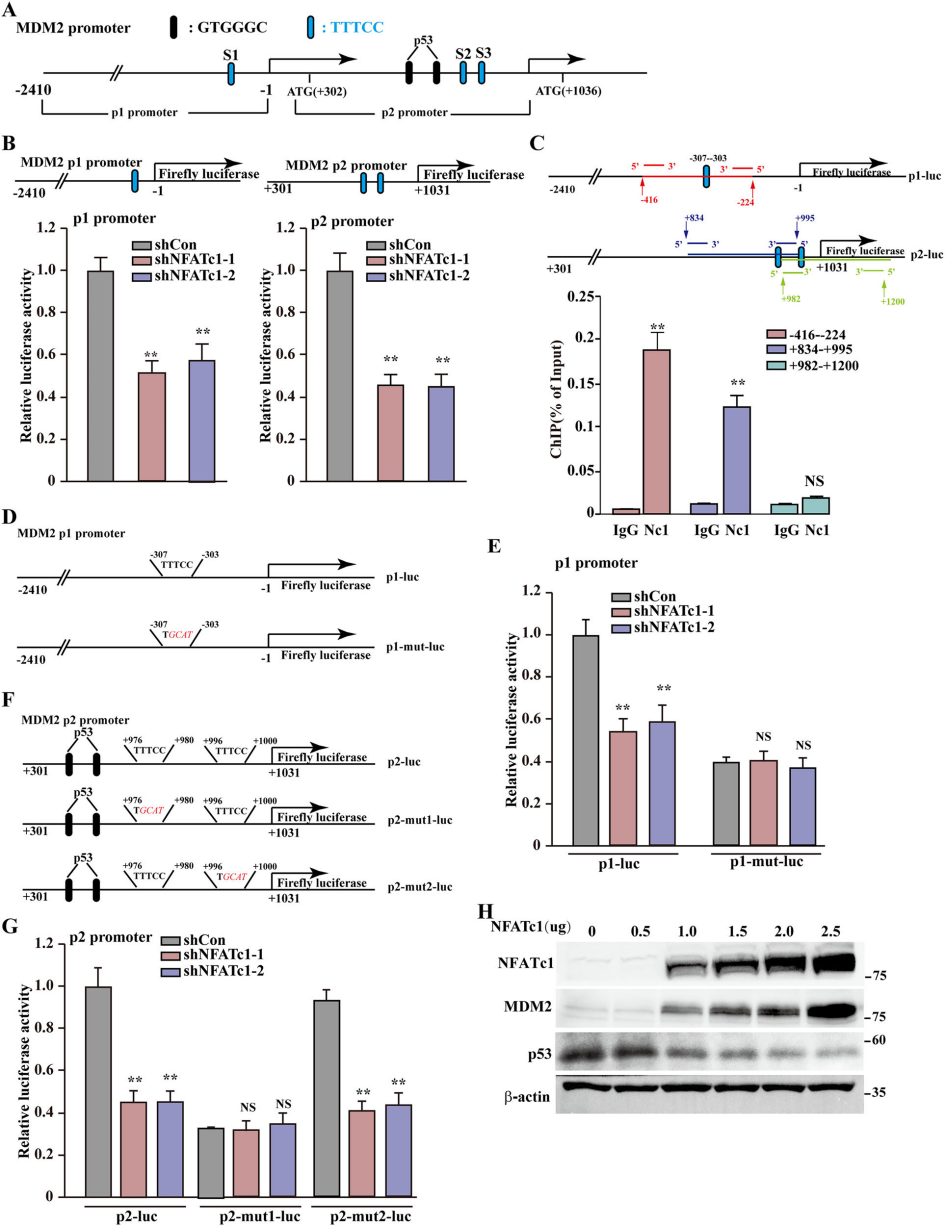
NFATc1 enhances MDM2 transcription through direct binding to both the p1 and p2 promoters of the MDM2 gene. **A** Schematic diagram of p1 and p2 promoters of MDM2 with two p53 binding sites (black) and three predicted NFATc1 binding sites (blue). **B** Schematic diagram and relative luciferase activities of MDM2 p1 promoter (left) and p2 promoter (right). **C** Binding of NFATc1 to the MDM2 promoter region was examined using chromatin immunoprecipitation assay with anti-NFATc1 antibody followed by qPCR in HCT116 cells. The predicted NFATc1 binding site on the MDM2 promoter and the location of primer sets used for qPCR for amplifying MDM2 promoter region with NFATc1 binding site are shown. **D** Schematic diagram of MDM2 p1 promoter reporter vector and MDM2 p1 promoter vector with mutated NFATc1 binding sites. Those mutated nucleotides are shown in italic red. **E** Relative luciferase activities of MDM2 p1 promoter and mutated p1 promoter. **F** Schematic diagram of MDM2 p2 promoter reporter vector and MDM2 p2 promoter vector with mutated NFATc1 binding sites. Those mutated nucleotides are shown in italic red. **G** Relative luciferase activities of MDM2 p2 promoter and mutated p2 promoter. **H** NFATc1, MDM2 and p53 protein accumulations in HCT116 cells transfected with indicated amounts of NFATc1 overexpression vector. Cells transfected with shCon or pcCon vector were used as control. β-Actin was used for qPCR normalization and as western blotting loading control. Quantitative data were expressed as mean ± SD. **p < 0.01; NS not significant.

Emerging evidence suggests NFATc1-mediated MDM2 regulation; however, its cis-regulatory architecture remains uncharacterized [29]. Based on predictions derived from the JASPAR database, we identified a binding site in the MDM2 promoter *p1* (−307 to −302; S1) and two binding sites in the *MDM2* promoter *p2* (+976 to +981; S2, and +996 to +1001; S3) (Fig. 5A). NFATc1 knockdown significantly reduced the transcriptional activity of both *MDM2 p1* and *p2* promoters (Fig. 5B). Consistently, in CRC HT29 and gastric cancer HGC-7901 cell lines, knockdown NFATc1 simultaneously suppressed the transcriptional activity of MDM2 (Fig. S5A, B). Subsequently, we performed a chromatin immunoprecipitation assay, which confirmed that NFATc1 binds to the −416 to −224 (S1) and +834 to +995 (S2) regions, but not to the +982 to +1200 (S3) region of the *MDM2* promoter (Fig. 5C). A luciferase reporter assay using p1-mut-luc revealed that mutations in the S1 binding site abolished the suppressive effect of *NFATc1* knockdown on wild-type MDM2-p1-luc (Fig. 5D, E). Similarly, mutations in the S2 binding site abolished the suppressive effect of *NFATc1* knockdown on wild-type MDM2-p2-luc. However, mutations at the S3 binding site did not exert significant effects (Fig. 5F, G). These results demonstrate that NFATc1 can bind to the S1 and S2 sites of the *MDM2* promoter, thereby upregulating its transcriptional activity.

p53 activates *MDM2* transcription via a feedback loop by binding to the *p2* promoter. Given that NFATc1 binds to both the *p1* and *p2* promoter regions downstream of the p53 binding site, NFATc1 binding to the *MDM2 p2* promoter region may interfere with the MDM2/p53 feedback loop, resulting in sustained MDM2 expression. To confirm this effect, we examined the dose-dependent inhibition of p53 accumulation with increasing levels of NFATc1 in HCT116 cells and observed a corresponding decrease in p53 protein accumulation, suggesting the absence of feedback regulation (Fig. 5H). Furthermore, we demonstrated that NFATc1 knockdown upregulates p53 target genes related to cell proliferation, an effect that was abolished upon p53 knockdown (Fig. S5C, S5D). Moreover, using p53-deficient HCT116 cells (HCT116 ^p53 null^), we found that NFATc1 knockdown resulted in a weaker suppression of cell proliferation compared to wild-type cells. Even when MDM2 was overexpressed in this context, no significant restoration of cell proliferation was observed (Fig. S5E–G). Collectively, these results suggest that the influence of the NFATc1/MDM2 pathway on colorectal cancer cell proliferation is primarily mediated through a p53-dependent mechanism.

### NFATc1 inhibitor demonstrates potent anti-tumor activity by targeting NFATc1 to downregulate NADK and MDM2

**Fig. 6:**
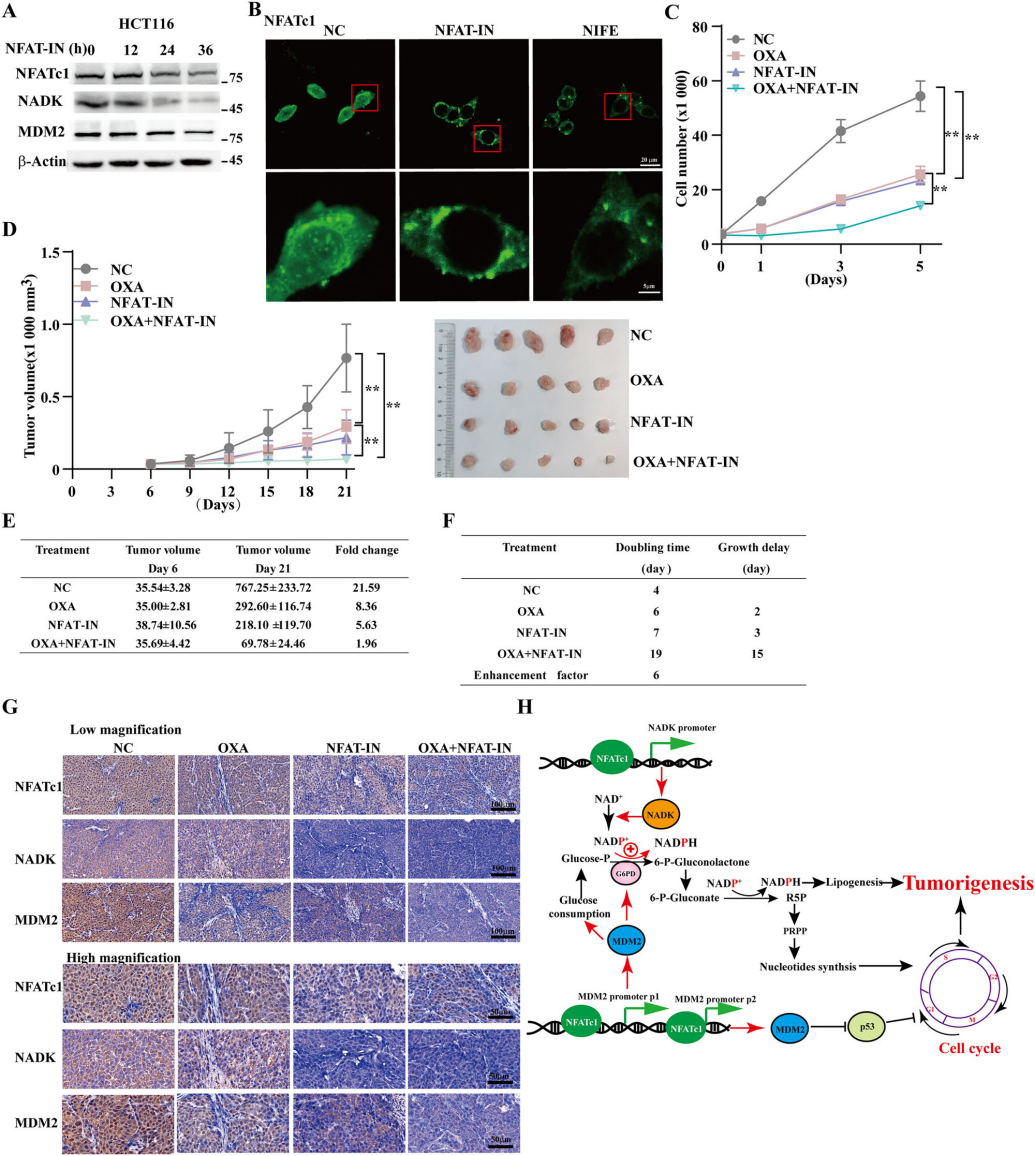
NFAT-IN inhibits CRC progression through NFATc1/NADK and NFATc1/MDM2 axis. **A** The protein expression level of NFATc1, NADK and MDM2 in HCT116 cells treated with 10 μM NFAT-IN for indicated time. **B** Cellular localization of NFATc1 was assessed by immunofluorescence after treatment with NFATc1 inhibitors (scale bars, 20 μm (top) and 5 μm (bottom)). **C** The total number of cells was determined following treatment with the indicated drug(s). **D** Volume and representative morphological images of xenografted tumors formed by HCT116 cells in BALB/c-nu/nu mice following indicated treatment at indicated time points (*n* = 5). **E** Fold-change of tumor volumes at day 21 compared to those at the starting point of the treatment (day 6). **F** Tumor growth delay and enhancement factor of combinatorial treatment of OXA and NFAT-IN. The enhancement factor was calculated as [(growth delay of combination) – (growth delay of NFAT-IN only)]/(growth delay of OXA only). **G** Immunohistochemistry staining showing the expression levels of NFATc1, NADK and MDM2 in tissue sections of xenografted tumors in BALB/c-nu/nu mice treated with indicated drugs. Representative of low-magnification (scale bars, 50 μm) and high magnification (scale bars, 25 μm) images. **H** Schematic diagram illustrating the molecular mechanism by which NFATc1 promotes CCR growth through enhancing the transcription of NADK and MDM2. β-Actin was used as western blotting loading control. Quantitative data were expressed as mean ± SD. **p < 0.01; NS not significant, OXA oxaliplatin.

The nuclear localization of NFATc1 is regulated by calcineurin. We used the NFAT-IN, which competitively binds to the calcineurin docking site for NFATc1, thereby blocking its nuclear import [16, 30]. Treatment with 10 μM NFAT-IN for 24 h led to a significant decrease in NFATc1 nuclear translocation and in NADK and MDM2 levels (Fig. 6A, B). Furthermore, we employed another NFATc1 inhibitor, NIFE [31], to further validate these findings. NIFE indirectly suppresses calcineurin activity by inhibiting calcium channels. Accordingly, 24 h treatment with NIFE resulted in a pronounced reduction in intracellular calcium levels, similarly preventing nuclear translocation of NFATc1 and downregulating the expression of NADK and MDM2 (Fig. 6B and S6A–C). We then employed a combination therapy utilizing NFATc1 inhibitor and oxaliplatin, a platinum analog approved for CRC treatment. The results showed that NFAT-IN and NIFE significantly inhibited CRC cell proliferation (Fig. 6C and S6D). Finally, we analyzed the effects of NFATc1 inhibitor on the NADK and MDM2 pathways in vivo and evaluated the therapeutic outcomes of combinatorial therapy with NFATc1 inhibitor and oxaliplatin. As evidenced by tumor volumes, treatment with oxaliplatin or NIFE alone inhibited tumor progression, and the combined treatment conspicuously enhanced this inhibitory effect (Fig. 6D and S6E). The combination of oxaliplatin and NFATc1 inhibitor significantly prolonged the doubling time (Fig. 6E and S6F). Accordingly, the synergistic interaction between oxaliplatin and NFATc1 inhibitor enhanced the antitumor efficacy compared to oxaliplatin alone, with an enhancement factor of 6 for NFAT-IN and 4 for NIFE (Fig. 6F and S6G). Furthermore, immunohistochemistry showed that this combination significantly inhibited NADK and MDM2 expression, leading to CRC growth inhibition. Furthermore, NFATc1 inhibitor significantly enhanced the anti-tumor efficacy of oxaliplatin (Fig. 6G and S6H).

In summary, our findings demonstrate that NFATc1 is significantly overexpressed in CRCs. It upregulates NADK expression, thereby increasing NADP^+^ production, activating PPP, and generating more NADPH to neutralize reactive oxygen species (ROS). Additionally, it enhances the synthesis of R5P, providing essential precursors for nucleic acid synthesis in rapidly proliferating cells. Furthermore, NFATc1 upregulates MDM2, which not only enhances metabolic reprogramming but also facilitates cell cycle progression, thereby further promoting tumor proliferation (Fig. 6H).

## Discussion

The PPP is a critical branching pathway of glycolysis that generates two key metabolic products, NADPH and R5P [7, 32]. Functionally, NADPH plays the following essential roles in tumor biology: (1) maintaining cellular redox homeostasis by preserving the reduced state of glutathione (GSH), thereby protecting tumor cells against oxidative damage [9, 33], and (2) functioning as a fundamental reducing equivalent in fuel biosynthetic processes, including de novo lipidogenesis and amino acid production [34]. Concurrently, R5P provides an indispensable ribose backbone required for nucleotide biosynthesis, which is a prerequisite for uncontrolled tumor proliferation [35]. Consequently, an enhanced PPP flux is a metabolic hallmark of rapidly proliferating malignancies.

Our mechanistic investigations demonstrated that NFATc1-mediated transcriptional upregulation of NADK drives the phosphorylation of NAD^+^ to NADP^+^, resulting in elevated NADP^+^ pools that allosterically activate G6PD, the rate-limiting enzyme in the PPP. This metabolic rewiring simultaneously fulfills the anabolic demands and reinforces the antioxidant defense systems of neoplastic cells. Furthermore, our findings establish that NFATc1 directly activates transcription from both the p1 and p2 promoters of MDM2—the central negative regulator of p53. Consequently, sustained MDM2 overexpression drives constitutive degradation of p53. Notably, p53 is known not only to orchestrate cell cycle arrest but also to antagonize tumor metabolic reprogramming by suppressing the Warburg effect and PPP through inhibition of GLUT family transporters, G6PD, HK2, among others [26–28, 36]. We confirmed that NFATc1 enhances lactate uptake and stimulates G6PD activity through upregulation of MDM2. Thus, NFATc1 promotes CRC tumorigenesis by concurrently activating the NADK and MDM2 pathways, which together disrupt essential cellular processes—including metabolic enzyme activity, NAD⁺/NADP⁺ redox homeostasis, and cell cycle control.

Furthermore, we utilized the NFATc1 inhibitor NFAT-IN to examine its impact on NADK and MDM2. NFAT-IN directly binds to the NFATc1-binding site on calcineurin to block NFATc1 dephosphorylation and nuclear translocation [16, 30]. Through this pharmacological approach, we further confirmed that NFATc1 facilitates the progression of CRC via NADK and MDM2. Furthermore, we evaluated the effects of combination therapy with NFAT-IN and oxaliplatin [37–39]. The combination of NFAT-IN and oxaliplatin enhanced the therapeutic effects of oxaliplatin and inhibited CRC cell proliferation.

NIFE is a dihydropyridine-L-type calcium channel blocker recommended by the World Health Organization for the clinical treatment of all types of hypertension [31, 40]. It indirectly inhibits calcineurin and the nuclear translocation of NFATc1 by blocking calcium ion channels. Since a case report in 2001 described the complete remission of a patient with metastatic CRC following high-dose NIFE treatment, its tumor-suppressive potential has attracted increasing attention [41]. However, the mechanisms underlying these tumor-suppressive effects remain unclear [42]. So, we utilized NIFE to inhibit NFATc1 and demonstrated that it exerts anti-tumor effects by suppressing both the NFATc1/NADK and NFATc1/MDM2 signaling pathways. Our findings uncover a novel molecular mechanism for the tumor-suppressive action of NIFE and offer new insights into its anti-tumor activity. Nevertheless, as a calcium channel blocker, NIFE lacks sufficient specificity for targeting NFATc1. Therefore, employing more specific NFATc1 inhibitors such as NFAT-IN, or developing novel and highly specific NFATc1 inhibitors, is essential for clarifying the therapeutic potential of NFATc1 inhibition in CRC treatment.

## Supplementary information

Supplementary Materials

Table S3 Source data and fold-change analysis results of differential metabolites between the NFATc1 knockdown group and the control group.

DATA SET Ct values of qPCR

The full length uncropped original western blots

## Data Availability

Data will be made available on request.

## References

[1] Siegel RL, Giaquinto AN, Jemal A. Cancer statistics, 2024. CA Cancer J Clin. 2024;74:12–49. 38230766 10.3322/caac.21820

[2] Dekker E, Tanis PJ, Vleugels JLA, Kasi PM, Wallace MB. Colorectal cancer. Lancet. 2019;394:1467–80. 31631858 10.1016/S0140-6736(19)32319-0

[3] Keum N, Giovannucci E. Global burden of colorectal cancer: emerging trends, risk factors and prevention strategies. Nat Rev Gastroenterol Hepatol. 2019;16:713–32. 31455888 10.1038/s41575-019-0189-8

[4] Shin AE, Giancotti FG, Rustgi AK. Metastatic colorectal cancer: mechanisms and emerging therapeutics. Trends Pharm Sci. 2023;44:222–36. 36828759 10.1016/j.tips.2023.01.003 PMC10365888

[5] Faubert B, Solmonson A, DeBerardinis RJ. Metabolic reprogramming and cancer progression. Science. 2020;368:eaaw5473. 10.1126/science.aaw5473 PMC7227780 32273439

[6] Fendt SM. 100 years of the Warburg effect: A cancer metabolism endeavor. Cell. 2024;187:3824–8. 39059359 10.1016/j.cell.2024.06.026

[7] Patra KC, Hay N. The pentose phosphate pathway and cancer. Trends Biochem Sci. 2014;39:347–54. 25037503 10.1016/j.tibs.2014.06.005 PMC4329227

[8] Meng Q, Zhang Y, Sun H, Yang X, Hao S, Liu B, et al. Human papillomavirus-16 E6 activates the pentose phosphate pathway to promote cervical cancer cell proliferation by inhibiting G6PD lactylation. Redox Biol. 2024;71:103108. 38457903 10.1016/j.redox.2024.103108 PMC10937312

[9] Tang Y, Li W, Qiu L, Zhang X, Zhang L, Miyagishi M, et al. The p52-ZER6/G6PD axis alters aerobic glycolysis and promotes tumor progression by activating the pentose phosphate pathway. Oncogenesis. 2023;12:17. 36977688 10.1038/s41389-023-00464-4 PMC10050210

[10] Xiao Y, Yu TJ, Xu Y, Ding R, Wang YP, Jiang YZ, et al. Emerging therapies in cancer metabolism. Cell Metab. 2023;35:1283–303. 37557070 10.1016/j.cmet.2023.07.006

[11] Rao A, Luo C, Hogan PG. Transcription factors of the NFAT family: regulation and function. Annu Rev Immunol. 1997;15:707–47. 9143705 10.1146/annurev.immunol.15.1.707

[12] Lucena PI, Faget DV, Pachulec E, Robaina MC, Klumb CE, Robbs BK, et al. NFAT2 Isoforms Differentially Regulate Gene Expression, Cell Death, and Transformation through Alternative N-Terminal Domains. Mol Cell Biol. 2016;36:119–31. 26483414 10.1128/MCB.00501-15 PMC4702591

[13] Macian F. NFAT proteins: key regulators of T-cell development and function. Nat Rev Immunol. 2005;5:472–84. 15928679 10.1038/nri1632

[14] Hasselluhn MC, Schlosser D, Versemann L, Schmidt GE, Ulisse M, Oschwald J, et al. An NFATc1/SMAD3/cJUN Complex Restricted to SMAD4-Deficient Pancreatic Cancer Guides Rational Therapies. Gastroenterology. 2024;166:298–312.e14.37913894 10.1053/j.gastro.2023.10.026

[15] Wang L, Mao X, Yu X, Su J, Li Z, Chen Z, et al. FPR3 reprograms glycolytic metabolism and stemness in gastric cancer via calcium-NFATc1 pathway. Cancer Lett. 2024;593:216841. 38614385 10.1016/j.canlet.2024.216841

[16] Gao J, Liu J, Lu J, Zhang X, Zhang W, Li Q, et al. SKAP1 Expression in Cancer Cells Enhances Colon Tumor Growth and Impairs Cytotoxic Immunity by Promoting Neutrophil Extracellular Trap Formation via the NFATc1/CXCL8 Axis. Adv Sci (Weinh). 2024;11:e2403430. 39269257 10.1002/advs.202403430 PMC11538704

[17] Klein AM, Biderman L, Tong D, Alaghebandan B, Plumber SA, Mueller HS, et al. MDM2, MDMX, and p73 regulate cell-cycle progression in the absence of wild-type p53. Proc Natl Acad Sci USA. 2021;118:e2102420118. 10.1073/pnas.2102420118 PMC8612355 34716260

[18] Oka SI, Titus AS, Zablocki D, Sadoshima J. Molecular properties and regulation of NAD(+) kinase (NADK). Redox Biol. 2023;59:102561. 36512915 10.1016/j.redox.2022.102561 PMC9763689

[19] Ilter D, Drapela S, Schild T, Ward NP, Adhikari E, Low V, et al. NADK-mediated de novo NADP(H) synthesis is a metabolic adaptation essential for breast cancer metastasis. Redox Biol. 2023;61:102627. 36841051 10.1016/j.redox.2023.102627 PMC9982641

[20] Lin W, Wang N, Wu S, Diao M, Huang Q, Li K, et al. NUAK1-Mediated Phosphorylation of NADK Mitigates ROS Accumulation to Promote Osimertinib Resistance in Non-Small Cell Lung Carcinoma. Cancer Res. 2024;84:4081–98. 39159134 10.1158/0008-5472.CAN-24-0249

[21] Miyagishi M, Taira K. Strategies for generation of an siRNA expression library directed against the human genome. Oligonucleotides. 2003;13:325–33. 15000823 10.1089/154545703322617005

[22] Huang C, Wu S, Ji H, Yan X, Xie Y, Murai S, et al. Identification of XBP1-u as a novel regulator of the MDM2/p53 axis using an shRNA library. Sci Adv. 2017;3:e1701383. 29057323 10.1126/sciadv.1701383 PMC5647124

[23] Huang C, Wu SR, Li WF, Herkilini A, Miyagishi M, Zhao HZ, et al. Zinc-finger protein p52-ZER6 accelerates colorectal cancer cell proliferation and tumour progression through promoting p53 ubiquitination. EBioMedicine. 2019;48:248–63. 31521611 10.1016/j.ebiom.2019.08.070 PMC6838388

[24] Wu S, Wang H, Li Y, Xie Y, Huang C, Zhao H, et al. Transcription Factor YY1 Promotes Cell Proliferation by Directly Activating the Pentose Phosphate Pathway. Cancer Res. 2018;78:4549–62. 29921695 10.1158/0008-5472.CAN-17-4047

[25] Huang Y, Che X, Wang PW, Qu X. p53/MDM2 signaling pathway in aging, senescence and tumorigenesis. Semin Cancer Biol. 2024;101:44–57. 38762096 10.1016/j.semcancer.2024.05.001

[26] Jiang P, Du W, Wang X, Mancuso A, Gao X, Wu M, et al. p53 regulates biosynthesis through direct inactivation of glucose-6-phosphate dehydrogenase. Nat Cell Biol. 2011;13:310–6. 21336310 10.1038/ncb2172 PMC3110666

[27] Schwartzenberg-Bar-Yoseph F, Armoni M, Karnieli E. The tumor suppressor p53 down-regulates glucose transporters GLUT1 and GLUT4 gene expression. Cancer Res. 2004;64:2627–33. 15059920 10.1158/0008-5472.can-03-0846

[28] Wu KK, Xu X, Wu M, Li X, Hoque M, Li GHY, et al. MDM2 induces pro-inflammatory and glycolytic responses in M1 macrophages by integrating iNOS-nitric oxide and HIF-1alpha pathways in mice. Nat Commun. 2024;15:8624. 39366973 10.1038/s41467-024-53006-w PMC11452520

[29] Hanaki S, Habara M, Tomiyasu H, Sato Y, Miki Y, Masaki T, et al. NFAT activation by FKBP52 promotes cancer cell proliferation by suppressing p53. Life Sci Alliance. 2024;7:e202302426. 10.26508/lsa.202302426 PMC11109481 38803221

[30] So T, Song JX, Sugie K, Altman A, Croft M. Signals from OX40 regulate nuclear factor of activated T cells c1 and T cell helper 2 lineage commitment. Proc Natl Acad Sci USA. 2006;103:3740–5. 16501042 10.1073/pnas.0600205103 PMC1450148

[31] Wu L, Lin W, Liao Q, Wang H, Lin C, Tang L, et al. Calcium Channel Blocker Nifedipine Suppresses Colorectal Cancer Progression and Immune Escape by Preventing NFAT2 Nuclear Translocation. Cell Rep. 2020;33:108327. 33113363 10.1016/j.celrep.2020.108327

[32] TeSlaa T, Ralser M, Fan J, Rabinowitz JD. The pentose phosphate pathway in health and disease. Nat Metab. 2023;5:1275–89. 37612403 10.1038/s42255-023-00863-2 PMC11251397

[33] Ying M, You D, Zhu X, Cai L, Zeng S, Hu X. Lactate and glutamine support NADPH generation in cancer cells under glucose deprived conditions. Redox Biol. 2021;46:102065. 34293554 10.1016/j.redox.2021.102065 PMC8321918

[34] Zhu Y, Gu L, Lin X, Liu C, Lu B, Cui K, et al. Dynamic Regulation of ME1 Phosphorylation and Acetylation Affects Lipid Metabolism and Colorectal Tumorigenesis. Mol Cell. 2020;77:138–49.e5. 31735643 10.1016/j.molcel.2019.10.015

[35] Xu JZ, Yang HK, Zhang WG. NADPH metabolism: a survey of its theoretical characteristics and manipulation strategies in amino acid biosynthesis. Crit Rev Biotechnol. 2018;38:1061–76. 29480038 10.1080/07388551.2018.1437387

[36] Liu Y, Gu W. The complexity of p53-mediated metabolic regulation in tumor suppression. Semin Cancer Biol. 2022;85:4–32. 33785447 10.1016/j.semcancer.2021.03.010 PMC8473587

[37] Shi Y, Niu Y, Yuan Y, Li K, Zhong C, Qiu Z, et al. PRMT3-mediated arginine methylation of IGF2BP1 promotes oxaliplatin resistance in liver cancer. Nat Commun. 2023;14:1932. 37024475 10.1038/s41467-023-37542-5 PMC10079833

[38] Pan Z, Zheng J, Zhang J, Lin J, Lai J, Lyu Z, et al. A Novel Protein Encoded by Exosomal CircATG4B Induces Oxaliplatin Resistance in Colorectal Cancer by Promoting Autophagy. Adv Sci (Weinh). 2022;9:e2204513. 36285810 10.1002/advs.202204513 PMC9762280

[39] Zeng K, Li W, Wang Y, Zhang Z, Zhang L, Zhang W, et al. Inhibition of CDK1 Overcomes Oxaliplatin Resistance by Regulating ACSL4-mediated Ferroptosis in Colorectal Cancer. Adv Sci (Weinh). 2023;10:e2301088. 37428466 10.1002/advs.202301088 PMC10477855

[40] Ferlinz J. Nifedipine in myocardial ischemia, systemic hypertension, and other cardiovascular disorders. Ann Intern Med. 1986;105:714–29. 3532894 10.7326/0003-4819-105-5-714

[41] Yang JL, Friedlander ML. Effect of nifedipine in metastatic colon cancer with DNA mismatch repair gene defect. Lancet. 2001;357:1767–8. 11403819 10.1016/S0140-6736(00)04892-3

[42] Liu G, Hu X, Premkumar L, Chakrabarty S. Nifedipine synergizes with calcium in activating the calcium sensing receptor, suppressing the expression of thymidylate synthase and survivin and promoting sensitivity to fluorouracil in human colon carcinoma cells. Mol Carcinog. 2011;50:922–30. 21374737 10.1002/mc.20752

