# Supplementary material for: NFATc1-mediated activation of the pentose phosphate pathway and cell cycle dysregulation collectively drive tumor progression
Source: Oncogenesis. 2025 Nov 7;14(1):39. doi: 10.1038/s41389-025-00581-2 (PMC12594948; doi:10.1038/s41389-025-00581-2)
Supplement: Supplementary file 1 — Supplementary Materials [file 41389_2025_581_MOESM1_ESM.docx]

**Supplementary Materials for**

**NFATc1-mediated activation of the pentose phosphate pathway and cell cycle dysregulation collectively drive tumor progression**

Suyang Zhang, Guangyao Xu, Tianyu Cao, Fei Yu, Moses Okotel, Mingyue Wu, Shourong Wu, Vivi Kasim, Can Huang

**This file includes:**

Supplementary Materials and Methods

Supplementary Figure S1–S6

Supplementary Table S1–S2

**Supplementary Materials and Methods**

**LC-MS/MS measurement**

1 × 10^7^ cells were collected with cold methanol: water solution (80% v/v) and centrifuged. The supernatant was collected and dried under nitrogen. The residue was reconstituted in solvent for LC-MS (Thermo Vanquish/Orbitrap Exploris 120) analysis. Mass spectrometry data were acquired in both positive and negative ionization modes with a high-resolution mass spectrometer. The mass range was set to m/z 50–1000. Raw LC-MS data were processed using specialized software (R XCMS（v3.12.0）) for peak detection, alignment, and annotation. Metabolite annotation was conducted by matching the experimental MS/MS spectra against publicly available and commercial databases, including HMDB, mzCloud, LipidMaps, MassBank and a custom metabolite database provided by Panomix Biomedical Tech Co., Ltd. (Suzhou, China). The molecular weight of metabolites was determined according to the m/z (mass-to-charge ratio) of parent ions in MS data. Molecular formula was predicted by ppm (parts per million) and adduct ion, and then matched with the database. At the same time, the MS/MS data from quantitative table of MS/MS data, were matched with the fragment ions and other information of each metabolite in the database, so as to realize the MS/MS identification of metabolites.

Data analysis

Both unsupervised (PCA) and supervised (PLS-DA; OPLS-DA) multivariate analyses were carried out with the R package ropls (v1.22.0) to distinguish sample groups. The statistical significance of intergroup differences was assessed based on p-values derived from appropriate hypothesis tests. Differential metabolite screening was subsequently carried out by integrating p-values, Variable Importance in Projection (VIP) scores from the OPLS-DA model, and fold change (FC) values. Metabolites with a p-value < 0.05 and a VIP > 1 were considered statistically significant and differentially expressed.

Pathway analysis

Pathway enrichment analysis was conducted using the hypergeometric test to identify functionally enriched metabolic pathways and perform topological analysis. Significantly altered metabolites identified in the metabolomic study were mapped to the KEGG pathway database for biological interpretation and higher-level systemic functional insights. The results, including key metabolites and their associated pathways, were visualized using the KEGG Mapper tool.

**Dual luciferase assay**

For dual luciferase assay, Cells were seeded into 24-well plates (8×10^4^ cells/well). 24 h later, the cells were co-transfected with the indicated vector. 24 h after co-transfection, the luciferase activities were then measured with the Dual Luciferase Assay System (Promega). Firefly luciferase activities were normalized with the corresponding Renilla luciferase activities.

**EdU incorporation assay**

Cell proliferation was assessed using the BeyoClick™ EdU Cell Proliferation Kit with Alexa Fluor 488 (Beyotime Biotechnology) following the manufacturer's protocol. Nuclei were counterstained with Hoechst 33342, and fluorescence images were acquired using a Zeiss AxioImager Z2 microscope equipped with appropriate filter sets.

**Quantification of total cell number**

Cells were prepared as described above. Cells were seeded into 96-well plates. The cell numbers were measured by Cell Counting-Lite 2.0 Luminescent Cell Viability Assay (Vazyme) at indicated time points.

**G6PD**

1×10⁶ cells were harvested, washed with PBS, and lysed via ultrasonic disruption. After centrifugation (12,000 ×g, 10 min, 4°C), the glucose-6-phosphate dehydrogenase (G6PD) activity in the supernatant was measured using a Human G6PD ELISA Kit (Boxbio) according to the manufacturer's protocol.

**PRPP concentration**

1×10⁶ cells were harvested and lysed via ultrasonic disruption. PRPP concentrations in the supernatant were determined using a Human Phosphoribosyl Pyrophosphate ELISA Kit (Boxbio) according to the manufacturer's instructions.

**Glucose consumption**

Cells were incubated for 24 hours, after which glucose levels in the medium were measured using a Glucose Assay Kit (O-toluidine method; Beyotime) following the manufacturer's protocol.

**Cell cycle analysis**

Cells were subjected for starvation for 24 h before being incubated further for 24 h under normal condition. Cells were then harvested and stained with propidium iodide (KeyGen Biotech, Nanjing, China). The percentages of the cells in each cell cycle phase were determined by flow cytometry.

**Chromatin immunoprecipitation (ChIP) assay**

Chromatin was immunoprecipitated using a ChIP Assay Kit (Beyotime Biotechnology) according to the manufacturer’s instructions. Briefly, cells were lysed and then chromatins were immunoprecipitated using anti-NFATc1 antibody, or normal rabbit IgG, and de-crosslinked for 4 hours at 65 °C. After being treated with 0.5 M EDTA, 1 M Tris (pH 6.5), and 20 mg/ml proteinase K, immunoprecipitated chromatin was then subjected to qPCR. Primer sequences for NADK: forward primer: 5'-CTGCTCTCCACCGCGATT-3'; reverse primer: 5'-ATGCGCGCTGCCGCGC-3'; primer sequences for MDM2: 416 to -242: forward primer: 5'-AGGTTGGCTCTGACTGTACC-3'; reverse primer: 5'-GATTCTCTTCCTCTGTGCGC-3'; +834 to +995: forward primer: 5'-GTCCAGATGAAGCTCCCAGA-3'; reverse primer: 5'-CAAGAAGCCCAGACGGAAAC-3'; +982 to +1200: forward primer: 5'-TGGCCATCTACAAGCAGTCA-3'; reverse primer: 5'-GGTACAGTCAGAGCCAACCT-3'

**Detection of calcium ions**

Ca^2+^ was detected with Fluo-4 Calcium Assay Kit (Beyotime Biotechnology). The cells in 24-well plates were washed with PBS and incubated with 200 μL Fluo-4 Staining Solution buffer containing 0.4 μL Fluo-4 AM and 0.4 μL Solubility Enhancer for 30 min, washed and incubated with PBS to stimulate Fluo-3 fluorescence at 488 nm. Concentration of calcium ions was detected using Calcium Colorimetric Assay Kit (Beyotime Biotechnology).

**Supplemental Figures and Legends**

**Supplementary Figure S1**


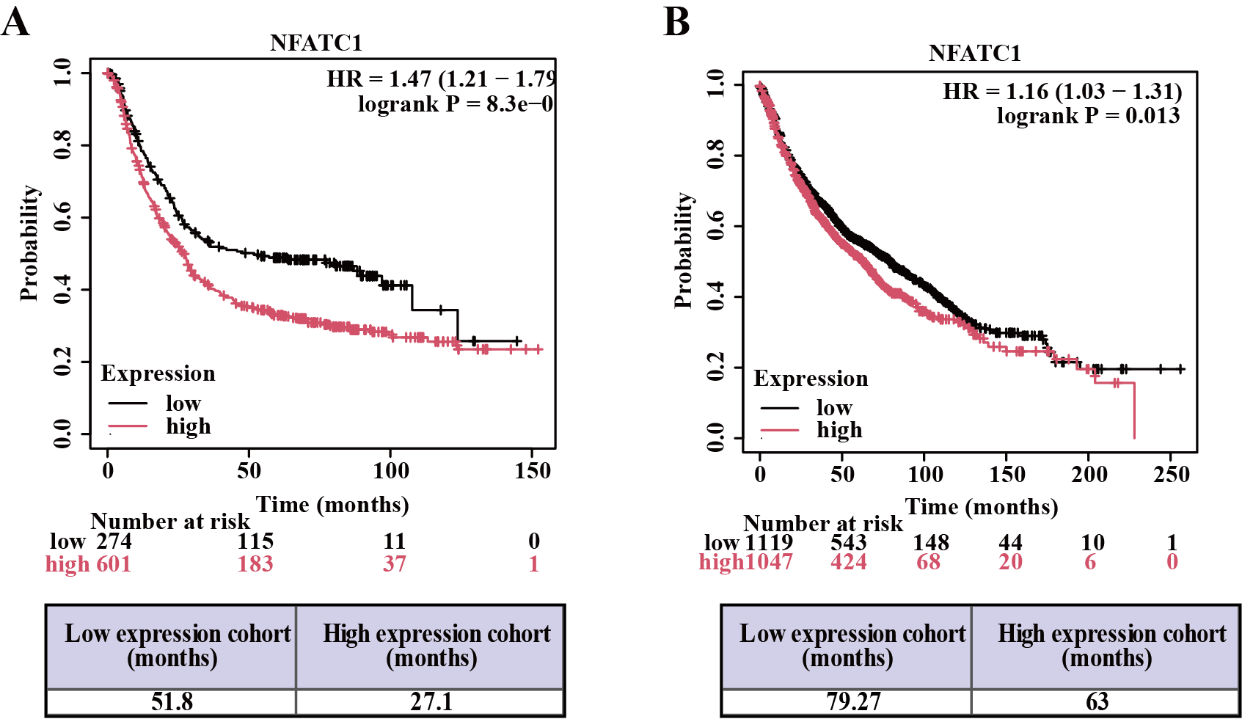


**Fig. S1 Kaplan-Meier survival plots of** **patients with either low (black) or high (red) NFATc1 expression in gastric cancer and lung cancer.** A the correlation between the expression of NFATc1 and survival plots in gastric cancer patients samples from GEO database. (n=875). B The correlation between the expression of NFATc1 and survival plots in lung cancer patients samples from GEO database. (n=2166).

**Supplementary Figure S2**


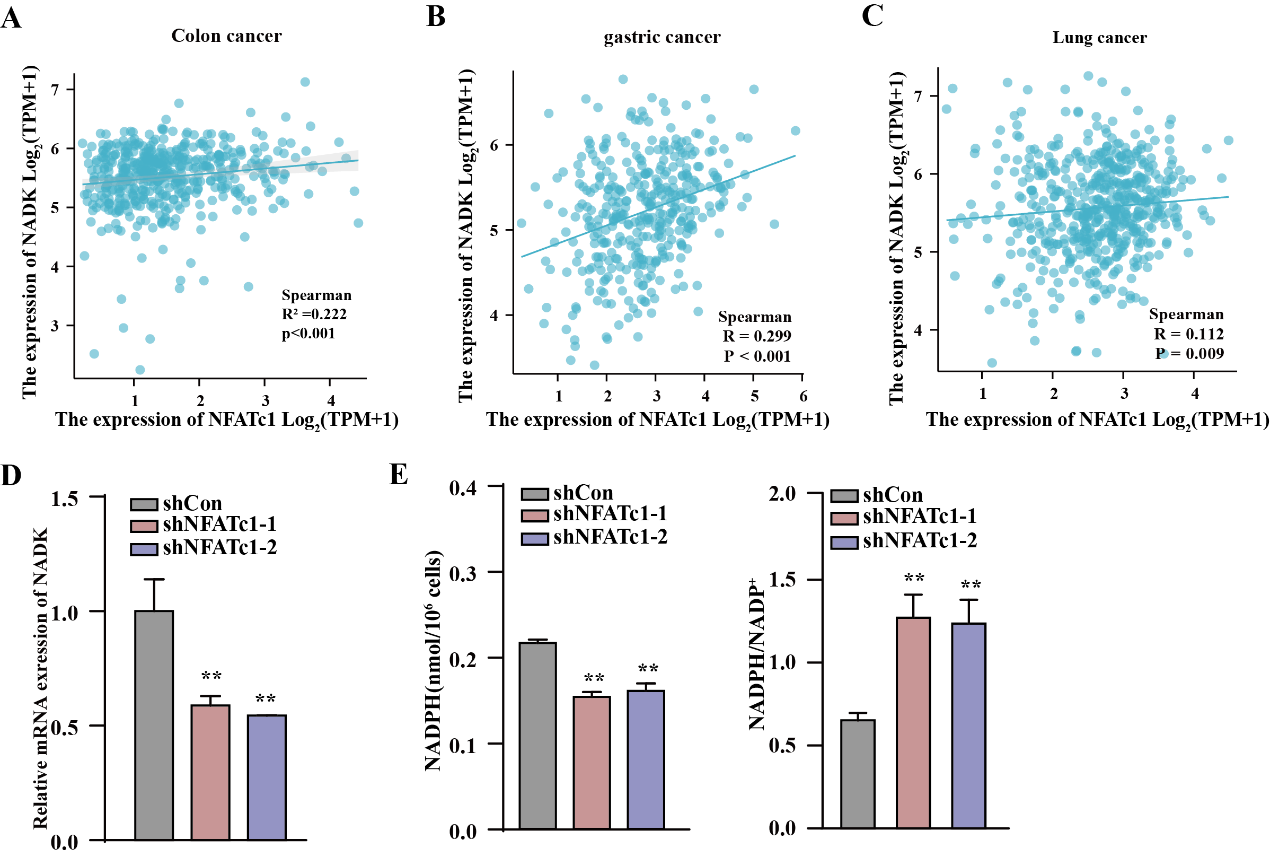


**Fig. S2 NFATc1 positively correlates with NADK expression and promotes the PPP.** A The correlation analysis of NFATc1 and NADK expression in CRC specimens (n=480). B The correlation between NFATc1 and NADK in tissue cells of gastric cancer patients (N=375). C The correlation between NFATc1 and NADK in tissue cells of lung cancer patients (N=539). D NADK mRNA expression level in HCT116 cells transfected with shRNA expression vectors targeting different sites of NFAFc1, as determined by qPCR. E The levels of NADPH and the NADPH to NADP+ ratio, following NFATc1 knockdown. β-Actin was used for qPCR normalization. Quantitative data were expressed as mean ±SD. **p<0.01.

**Supplementary Figure S3**

**
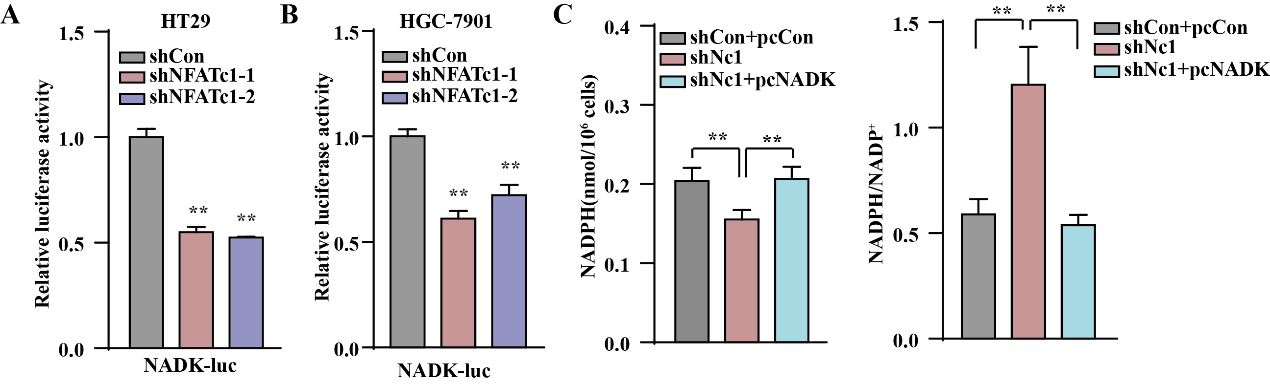
**

**Fig. S3 NFATc1 stimulates the PPP through increased transcription of NADK.** A–B NFATc1 enhances the transcriptional activation of NADK in HT29 (A) and HGC-7901 (B) cells. C NADPH levels and NADPH/NADP⁺ ratio in HCT116 cells following NFATc1 knockdown and NADK overexpression. Quantitative data were expressed as mean ±SD. ⁎⁎p<0.01.

**Supplementary Figure S4**


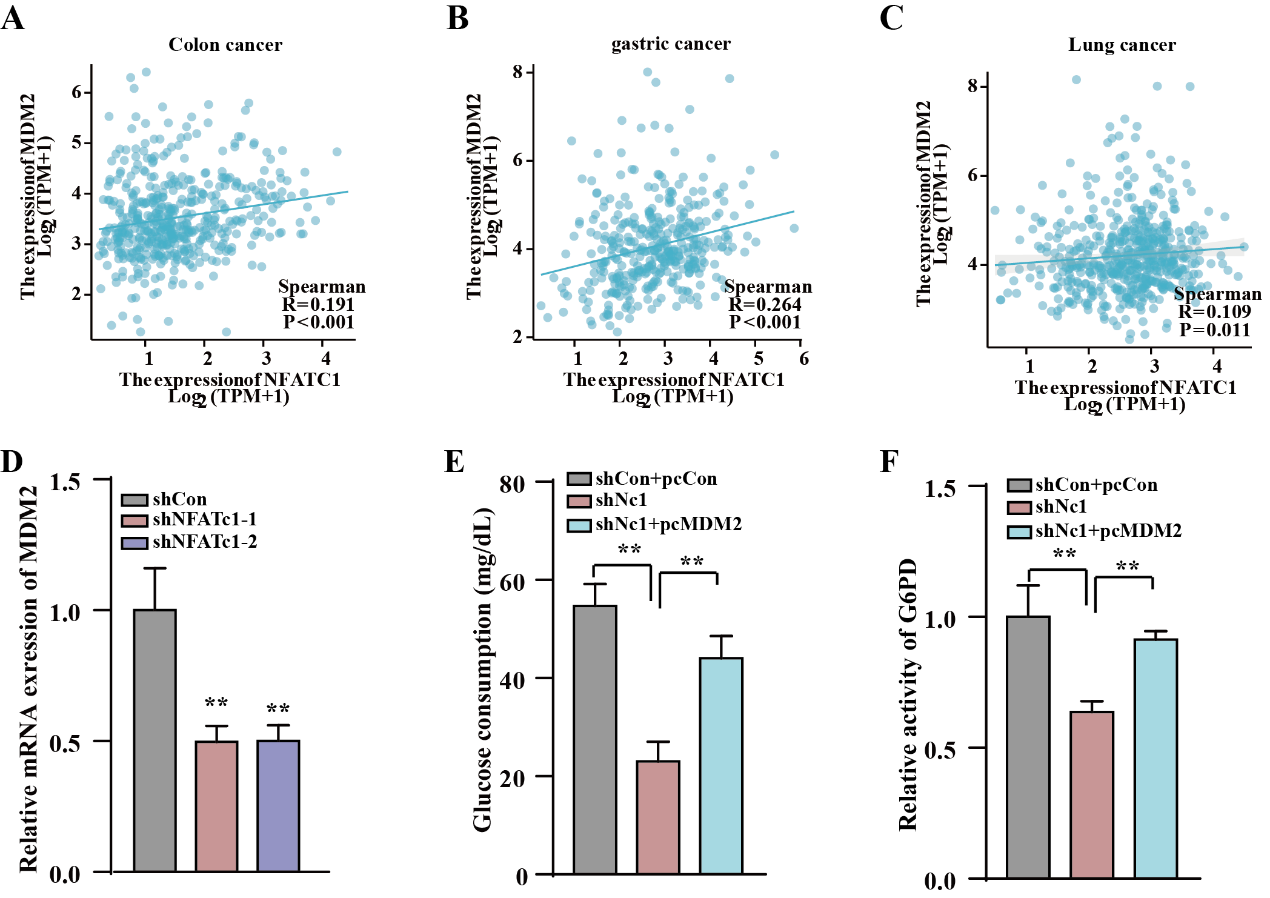


**Fig. S4 NFATc1 positively correlates with MDM2 and promotes metabolic reprogramming.** A The correlation between NFATc1 and MDM2 in tissue cells of CRC patients (N=480). B The correlation between NFATc1 and MDM2 in tissue cells of gastric cancer patients (N=375). C The correlation between NFATc1 and MDM2 in tissue cells of lung cancer patients (N=539). D MDM2 mRNA expression level in HCT116 cells transfected with shRNA expression vectors targeting different sites of NFAFc1, as determined by qPCR. E Glucose consumption following NFATc1 knockdown and MDM2 overexpression. F Relative activity of G6PD following NFATc1 knockdown and MDM2 overexpression. Quantitative data were expressed as mean ±SD. ⁎⁎p<0.01.

**Supplementary Figure S5**


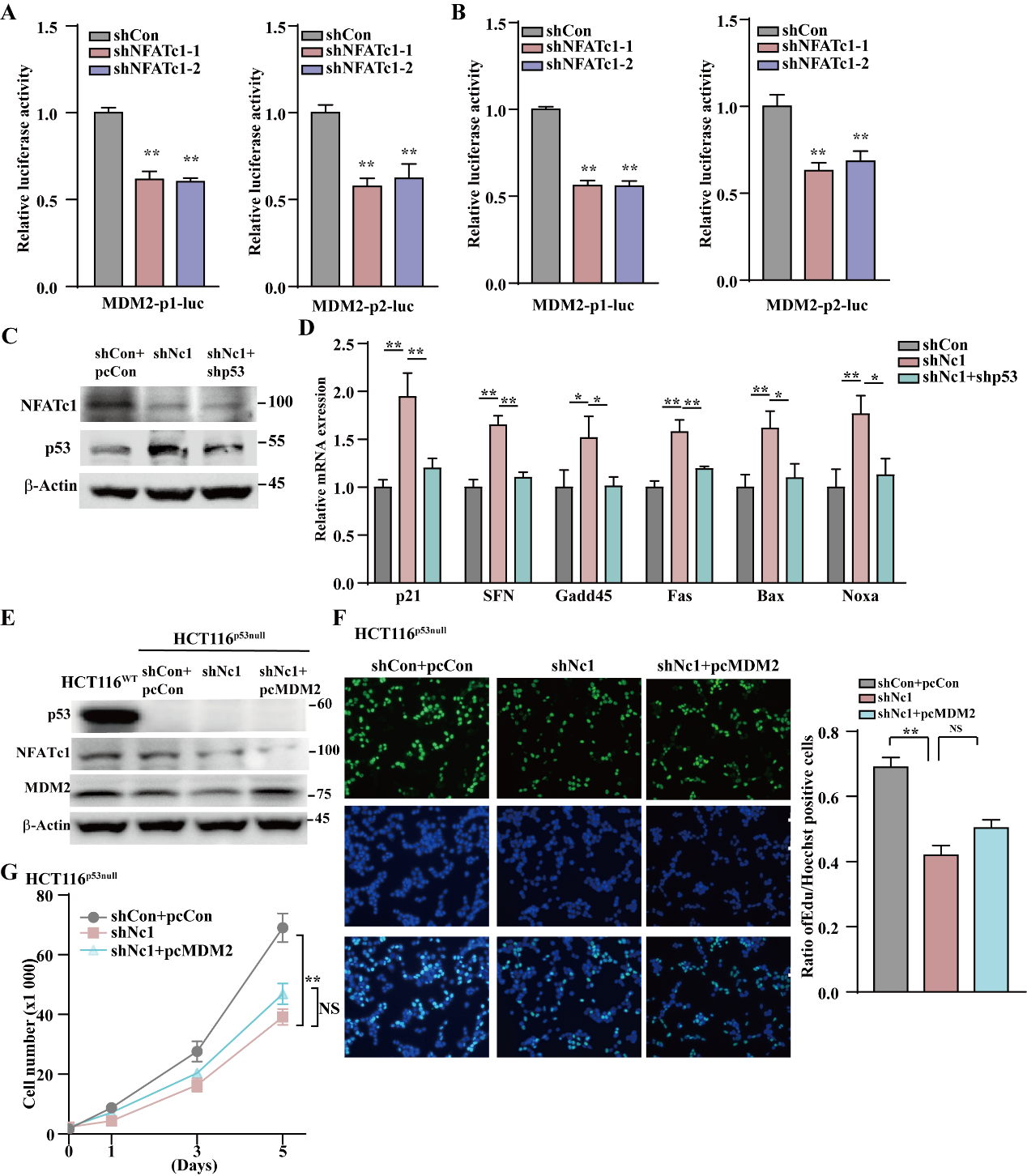


**Fig. S5** **The NFATc1/MDM2 pathway drives CRC proliferation through p53.** A–B NFATc1 enhances the transcriptional activation of MDM2 in HT29 (A) and HGC-7901 (B) cells. C–D The effect of shNFATc1 and shp53 co-transfection on the mRNA expression of p53 downstream factors was conducted by qPCR. E Protein expression level of p53, NFATc1 and MDM2 in HCT116 p53^null^ cells transfected with indicated shRNA expression vectors and overexpression vectors. F Representative images of proliferating cells, as identified by the EdU incorporation assay. Hoechst was used to stain nuclei (scale bars, 100 μm). G The total number of cells was determined following NFATc1 knockdown and MDM2 overexpression at the indicated time points. Quantitative data were expressed as mean ±SD. ⁎⁎p<0.01; NS: not significant; Nc1: NFATc1.

**Supplementary Figure S6**

**
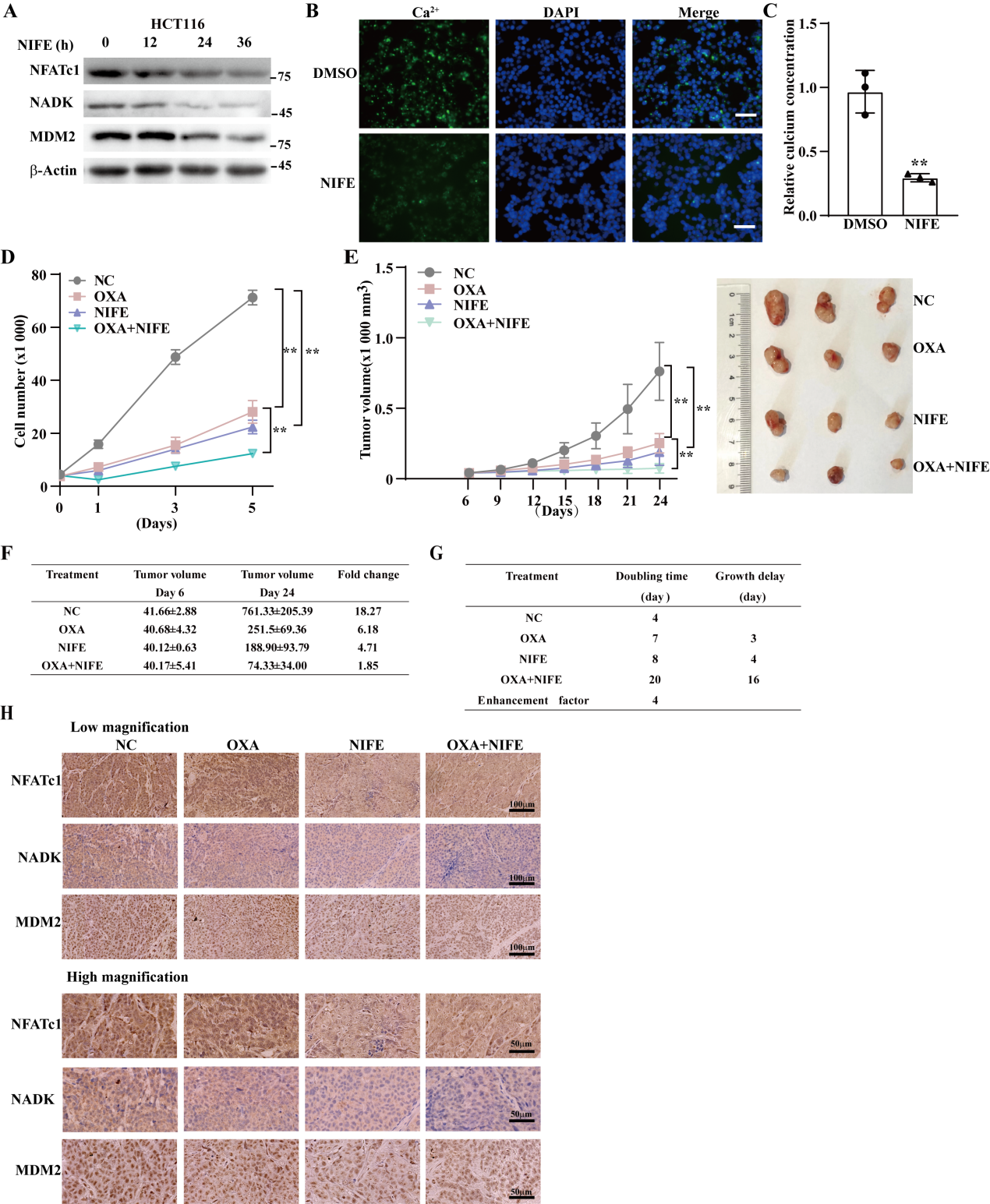
**

**Fig. S6** **NIFE inhibits CRC progression through NFATc1/NADK and NFATc1/MDM2 axis.** A The protein expression level of NFATc1, NADK and MDM2 in HCT116 cells treated with 10 μM nifedipine for indicated time. B The representative images of Ca^2+^ in cells treated with nifedipine for 12 h were detected using Fluo-4 Calcium Assay Kit (scale bars, 50 μm). C Concentration of Ca^2+^ in cells treated with nifedipine for 12 h were detected by Calcium Colorimetric Assay Kit. D The total number of cells was determined following treatment with the indicated drug(s). E Volume of xenografted tumors formed by HCT116 cells in BALB/c-nu/nu mice following indicated treatment at indicated time points (n=3). F Fold-change of tumor volumes at day 24 compared to those at the starting point of the treatment (day 6). G Tumor growth delay and enhancement factor of combinatorial treatment of OXA and NIFE. The enhancement factor was calculated as [(growth delay of combination) – (growth delay of NIFE only)]/(growth delay of OXA only). H Immunohistochemistry staining showing the expression levels of NFATc1, NADK and MDM2 in tissue sections of xenografted tumors in BALB/c-nu/nu mice treated with indicated drugs. Representative of low-magnification (scale bars, 50 μm) and high magnification (scale bars, 25 μm) images. Quantitative data were expressed as mean ±SD. ⁎⁎p<0.01; NS: not significant; OXA: oxaliplatin; NIFE: nifedipine.

**Supplementary Table S1**

Primer pairs used for gene quantification by quantitative PCR.

| **Gene** | **RefSeq No.** | **Forward** | **Reverse** |
| --- | --- | --- | --- |
| *p21* | NM_000389.4 | TCACTGTCTTGTACCCTTGTGC | GGCGTTTGGAGTGGTAGAAA |
| *NADK* | NM_023018.5 | GCAGCGAACGGCATCAGT | GGGGTGGTTGTAACTCCAGG |
| *MDM2* | NM_002392.5 | TGTTGGTGCACAAAAAGACA | CACGCCAAACAAATCTCCTA |
| *NFATc1* | NM_172390.3 | CAAGCCGAATTCTCTGGTGG | CTGGTACTGGCTTCGCTTTC |
| *p53* | NM_000546.5 | AGGCCTTGGAACTCAAGGAT | GGTAGACTGACCCTTTTTGGAC |
| *SFN* | NM_006142.5 | AGACAGCACCCTCATCATGC | AGCCCTTTGGAGCAAGAACA |
| *Gadd45* | NM_001924.4 | TCACTGTCTTGTACCCTTGTGC | GGCGTTTGGAGTGGTAGAAA |
| *Fas* | NM_000043 | ACTGTGACCCTTGCACCAAA | AGACAAAGCCACCCCAAGTT |
| *Bax* | NM_001291428 | TGATGGACGGGTCCGGG | GGAAAAAGACCTCTCGGGGG |
| *Noxa* | NM_001382616.1 | CGAGGAACAAGTGCAAGTAGC | ACGTGCACCTCCTGAGAAAA |
| *β-Actin* | NM_001101.3 | CGAGCGCGGCTACAGCTT | TCCTTAATGTCACGCACGATTT |

**Supplementary Table S2**

Antibodies used for western blotting and immunohistochemistry.

| **Antibody** | **Maker** | **Product number** |
| --- | --- | --- |
| NFATc1 | Santa Cruz Biotechnology | sc-7294 |
| NADK | Proteintech | 15548-1-AP |
| β-Actin | Proteintech | 60008-1- Ig |
| GAPDH | Proteintech | 60004-1-Ig |
| p21 | Proteintech | 10355-1-AP |
| p53 | Proteintech | 10442-1-AP |
| MDM2 | Santa Cruz Biotechnology | sc-965 |
| Goat Anti-Rabbit IgG | ZSGB-BIO | ZB2301 |
| Goat Anti-Mouse IgG | ZSGB-BIO | ZB2305 |
