# Supplementary material for: NFATc1-mediated activation of the pentose phosphate pathway and cell cycle dysregulation collectively drive tumor progression
Source: Oncogenesis. 2025 Nov 7;14(1):39. doi: 10.1038/s41389-025-00581-2 (PMC12594948; doi:10.1038/s41389-025-00581-2)
Supplement: Supplementary file 3 — DATA SET Ct values of qPCR [file 41389_2025_581_MOESM3_ESM.pdf]

Fig. 1D (N=30)	Ct Actin						
**Sample Num.**		**Nomal**			**Tumor**		
**1**	**actin**	18.5	18.55	18.54	18.19	18.11	18.12
**2**	**actin**	18.35	18.54	18.33	18.02	18.02	18.31
**3**	**actin**	18.62	18.66	18.56	17.86	17.89	17.92
**4**	**actin**	18.19	18.37	18.14	18.28	18.22	18.23
**5**	**actin**	18.02	18.01	18.03	18.06	18.05	18.11
**6**	**actin**	17.86	18.02	18.96	17.95	18.02	18.11
**7**	**actin**	18.28	18.22	18.32	18.5	18.43	18.57
**8**	**actin**	18.06	18.11	18	18.35	18.33	18.46
**9**	**actin**	17.95	18.01	18.11	18.62	18.61	18.57
**10**	**actin**	18.5	18.65	18.61	18.19	18.15	18.21
**11**	**actin**	18.35	18.41	18.29	18.02	18.11	18.06
**12**	**actin**	18.62	18.65	18.61	17.86	18.01	17.89
**13**	**actin**	18.19	18.21	18.31	18.28	18.32	18.22
**14**	**actin**	18.02	18.03	18.04	18.06	18.02	18.04
**15**	**actin**	17.86	18.01	17.92	17.95	17.99	18.04
**16**	**actin**	18.28	18.22	18.31	18.5	18.51	18.46
**17**	**actin**	18.06	18.06	18.11	18.35	18.33	18.28
**18**	**actin**	17.95	18	18.12	18.62	18.56	18.63
**19**	**actin**	18.5	18.54	18.49	18.19	18.22	18.17
**20**	**actin**	18.35	18.41	18.46	18.02	18.08	18.06
**21**	**actin**	18.62	18.62	18.59	17.86	17.84	17.86
**22**	**actin**	18.19	18.21	18.31	18.28	18.22	18.31
**23**	**actin**	18.02	18.11	18.23	18.06	18	18.05
**24**	**actin**	17.86	18.11	18.02	17.95	18.01	18.07
**25**	**actin**	18.28	18.22	18.26	18.5	18.46	18.52
**26**	**actin**	18.06	18.06	18.04	18.35	18.33	18.41
**27**	**actin**	17.95	18	18.12	18.62	18.58	18.56
**28**	**actin**	18.5	18.23	18.21	18.19	18.11	18.17
**29**	**actin**	18.35	18.31	18.26	18.02	18.01	18.02
**30**	**actin**	18.62	18.66	18.54	17.86	17.96	18.01

Fig. 1D	Ct NFATc1						
Sample Num.		Nomal			Tumor		
1	NFATc1	22.50	22.83	22.79	21.68	21.84	21.50
2	NFATc1	23.51	23.99	23.75	22.19	22.44	22.37
3	NFATc1	24.93	25.28	25.15	22.20	22.48	22.15
4	NFATc1	23.59	24.06	23.80	22.63	22.82	22.46
5	NFATc1	23.90	24.19	24.18	21.36	21.59	21.31
6	NFATc1	23.51	23.96	24.88	22.20	22.52	22.25
7	NFATc1	22.63	22.84	22.92	22.38	22.56	22.34
8	NFATc1	23.89	24.24	24.10	20.85	21.06	20.85
9	NFATc1	22.57	22.91	22.98	22.14	22.38	21.99
10	NFATc1	24.68	25.14	25.07	22.53	22.74	22.44
11	NFATc1	24.26	24.62	24.47	21.90	22.24	21.83
12	NFATc1	23.57	23.89	23.82	21.59	21.99	21.52
13	NFATc1	25.40	25.73	25.80	22.83	23.13	22.66
14	NFATc1	23.11	23.40	23.38	20.26	20.45	20.14
15	NFATc1	22.14	22.56	22.44	21.03	21.31	21.02
16	NFATc1	21.55	21.75	21.82	20.79	21.04	20.65
17	NFATc1	21.87	22.13	22.16	20.79	21.00	20.62
18	NFATc1	23.46	23.80	23.90	23.05	23.25	22.95
19	NFATc1	24.62	24.96	24.88	23.05	23.34	22.92
20	NFATc1	23.47	23.82	23.84	22.86	23.17	22.78
21	NFATc1	23.80	24.10	24.04	21.60	21.82	21.49
22	NFATc1	23.70	24.02	24.09	23.17	23.37	23.09
23	NFATc1	25.34	25.74	25.84	24.70	24.92	24.57
24	NFATc1	24.92	25.48	25.36	24.69	25.03	24.69
25	NFATc1	22.66	22.88	22.89	21.78	21.98	21.69
26	NFATc1	24.12	24.42	24.37	22.89	23.12	22.83
27	NFATc1	24.57	24.92	25.01	23.84	24.07	23.67
28	NFATc1	23.81	23.83	23.78	23.37	23.55	23.24
29	NFATc1	23.27	23.52	23.44	22.71	22.95	22.60
30	NFATc1	24.07	24.41	24.26	21.89	22.24	21.94

**Fig. 1G-Three Times(N=3, n=3)**		
**1**	**Ct NFATc1**	**Ct Actin**
**shCon**	21.02	18.91
	21.03	18.91
	21.05	18.91
**shNFATc1-1**	22.56	18.85
	22.61	18.84
	22.68	18.83
**shNFATc1-2**	22.31	18.88
	22.31	18.78
	22.36	18.91
**shNFATc1-3**	22.15	18.52
	22.09	18.53
	22.08	18.55
**2**		
**shCon**	21.35	18.78
	21.34	18.77
	21.36	18.69
**shNFATc1-1**	22.56	18.91
	22.68	18.88
	22.69	18.86
**shNFATc1-2**	22.77	18.88
	22.76	18.88
	22.74	18.86
**shNFATc1-3**	22.23	18.43
	22.32	18.42
	22.27	18.51
**3**		
**shCon**	21.22	18.86
	21.23	18.55
	21.25	18.49
**shNFATc1-1**	22.65	18.77
	22.75	18.78
	22.71	18.79
**shNFATc1-2**	22.65	18.91
	22.59	18.88
	22.56	18.96
**shNFATc1-3**	22.13	18.76
	22.14	18.71
	22.14	18.75

Fig.S2D-Three Times(N=3, n=3)		
1	Ct NADK	Ct Actin
shCon	23	18.91
	23.4	18.91
	23.2	18.91
shNFATc1-1	24.1	18.85
	23.89	18.84
	24.01	18.83
shNFATc1-2	24.22	18.88
	24.13	18.78
	24.32	18.91
2		
shCon	23.12	18.78
	23.41	18.77
	23.32	18.69
shNFATc1-1	24.12	18.91
	24.16	18.88
	24.09	18.86
shNFATc1-2	24.23	18.88
	24.22	18.88
	24.25	18.86
3		
shCon	23.31	18.86
	23.34	18.55
	23.29	18.49
shNFATc1-1	24.12	18.77
	24.15	18.78
	24.16	18.79
shNFATc1-2	24.23	18.91
	24.31	18.88
	24.29	18.96

**Fig. S4D-Three Times(N=3, n=3)**		
**1**	**Ct MDM2**	**Ct Actin**
**shCon**	22.12	18.91
	22.16	18.91
	22.15	18.91
**shNFATc1-1**	23.09	18.85
	23.05	18.84
	23.01	18.83
**shNFATc1-2**	23.11	18.88
	23.04	18.78
	23.03	18.91
**2**		
**shCon**	21.49	18.78
	21.37	18.77
	21.72	18.69
**shNFATc1-1**	22.79	18.91
	22.81	18.88
	22.89	18.86
**shNFATc1-2**	22.72	18.88
	22.77	18.88
	22.85	18.86
**3**		
**shCon**	21.11	18.86
	21.17	18.55
	30.18	18.49
**shNFATc1-1**	22.59	18.77
	22.64	18.78
	22.64	18.79
**shNFATc1-2**	22.79	18.91
	22.73	18.88
	22.85	18.96

Fig. S5D-Three Times(N=3, n=3)							
1	Ct Actin	Ct p21	Ct SNF	Ct Gadd45	Ct Fas	Ct Bax	Ct Noxa
**NC**	20.13	21.22	21.02	22.68	24.15	25.32	22.86
	20.41	21.23	21.03	22.69	24.3	25.42	22.97
	20.19	21.31	21.01	22.67	24.16	25.53	22.97
**shNFATc1**	19.69	19.95	19.85	21.81	23.15	24.36	21.69
	19.65	19.86	19.74	21.65	23	24.33	21.89
	19.75	19.91	19.68	21.67	23.14	24.26	21.66
**shNFATc1+shp53**	19.55	20.15	20.16	22.41	23.18	24.62	22.04
	19.54	20.24	20.1	22	23.35	24.72	22.04
	19.43	20.21	20.06	22.32	23.15	24.45	22.16
2							
**NC**	19.31	20.36	20.23	21.72	23.36	24.65	22.59
	19.32	20.33	20.2	21.89	23.33	24.84	22.68
	19.46	20.51	20.38	22.07	23.51	24.89	22.49
**shNFATc1**	19.65	19.65	19.72	21.41	22.81	24.33	21.99
	19.64	19.55	19.69	21.51	22.92	24.11	21.93
	19.59	19.46	19.93	21.43	23.06	24.15	21.96
**shNFATc1+shp53**	19.65	20.46	20.33	22.01	23.24	24.89	22.59
	19.55	20.35	20.22	22.11	23.35	24.76	22.77
	19.59	20.55	20.45	22.05	23.29	24.69	22.45
3							
**NC**	18.69	19.78	19.23	21.54	22.36	24.05	21.78
	18.59	20.01	19.24	21.57	22.49	24.25	21.61
	18.49	19.65	19.35	21.51	22.48	24.11	21.46
**shNFATc1**	18.55	18.75	18.73	20.72	21.74	23.45	20.59
	18.47	18.78	18.45	20.64	21.64	23.32	20.42
	18.43	18.69	18.23	20.85	21.59	23.31	20.63
**shNFATc1+shp53**	18.65	19.55	19.25	21.21	22.45	24.16	21.54
	18.49	19.46	19.22	21.22	22.32	24.13	21.14
	18.71	19.61	19.2	21.34	22.1	24	21.65
**NIFE-36**	20.13	21.22	21.02	22.68	24.15	25.32	22.86
	20.41	21.23	21.03	22.69	24.3	25.42	22.97
	20.19	21.31	21.01	22.67	24.16	25.53	22.97
