# Supplementary figures and images for: NFATc1-mediated activation of the pentose phosphate pathway and cell cycle dysregulation collectively drive tumor progression
Source: Oncogenesis. 2025 Nov 7;14(1):39. doi: 10.1038/s41389-025-00581-2 (PMC12594948; doi:10.1038/s41389-025-00581-2)

Fig. 1E

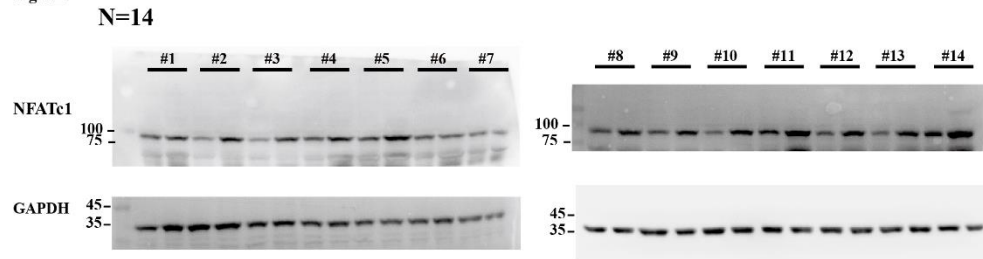

Fig. 1H

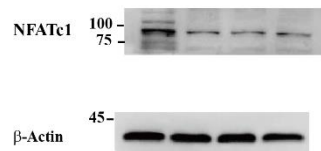

Fig. 2B

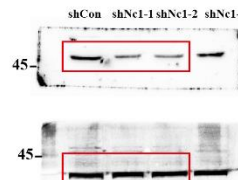

Fig. 3H

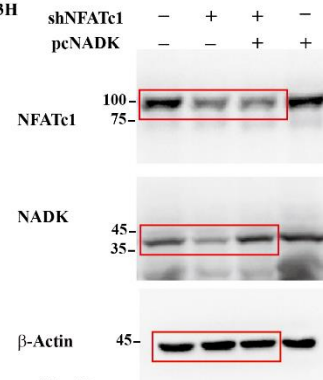

Fig. 4A

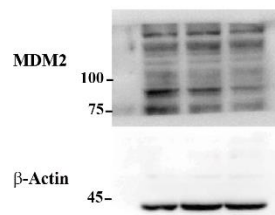

Fig. 4B

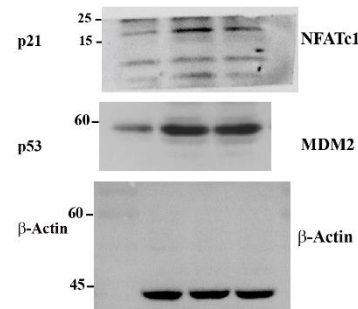

Fig. 4D

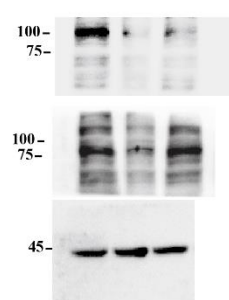

Fig. 4H

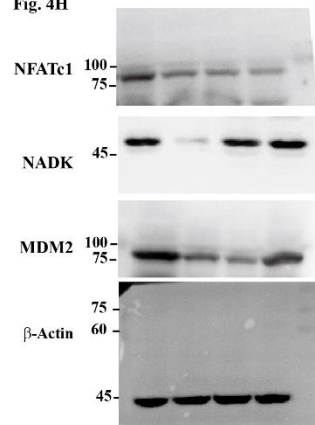

Fig. 5H

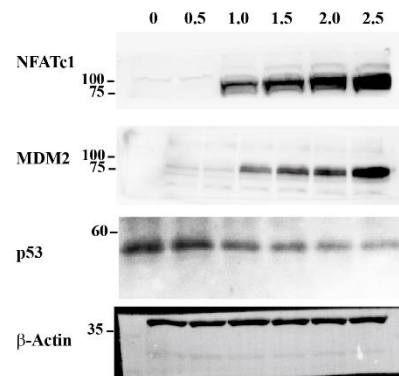

Fig. 6A

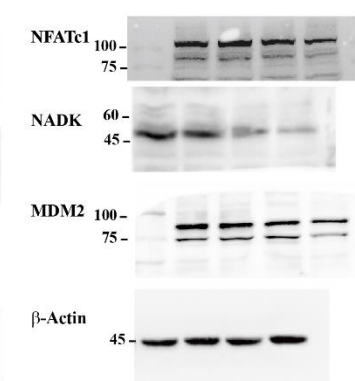

SSC

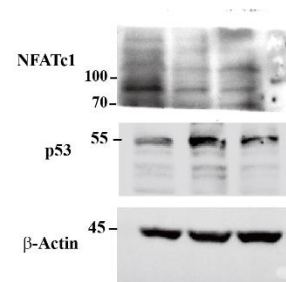

SSE

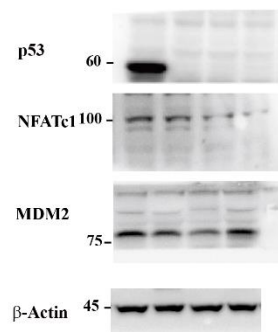

Fig. S6A

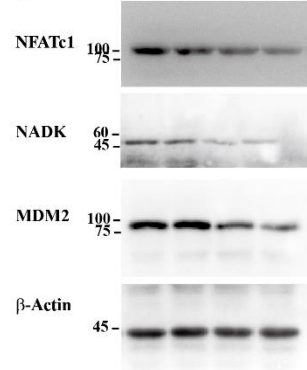

Supplement: Supplementary file 4 — The full length uncropped original western blots [file 41389_2025_581_MOESM4_ESM.pdf]
